# Molecular characterization reveals three *Neopestalotiopsis* species causing strawberry disease outbreaks in Spain

**DOI:** 10.3389/fpls.2026.1830265

**Published:** 2026-05-11

**Authors:** Patricia López, Ana M. Pastrana, Marcus V. Marin, Celia Borrero, Natalia A. Peres, Manuel Avilés

**Affiliations:** 1 Departamento de Agronomía, ETSIA-Universidad de Sevilla, Sevilla, Spain; 2 University of California Agriculture and Natural Resources, Cooperative Extension, Holtville, CA, United States; 3 Plant Pathology Department, Gulf Coast Research and Education Center, University of Florida, Wimauma, FL, United States

**Keywords:** emerging pathogens, *Neopestalotiopsis iranensis*, *Neopestalotiopsis rosae*, phylogenetic analysis, strawberry diseases

## Abstract

The emergence of new fungal diseases is one of the major challenges affecting strawberry production worldwide. Among them, *Pestalotiopsis*-like species have been reported in multiple countries as causal agents of fruit rot, leaf spot, root rot, and crown rot. In recent years, these pathogens have caused numerous disease outbreaks in Spanish strawberry fields, threatening industry sustainability and requiring accurate identification for effective disease management. Phylogenetic characterization, based on internal transcribed spacer (ITS), elongation factor (TEF), and β-tubulin (TUB) regions, led to the identification of phylogenetically distinct groups in Spain: *Neopestalotiopsis rosae*, *Neopestalotiopsis* sp., and *N. iranensis*. Pathogenicity of the isolates was confirmed by inoculating two representative isolates of each species onto three strawberry cultivars, ‘Rociera FNM’, ‘Palmeritas’, and ‘Plared 15105’. Differences in aggressiveness were observed on leaf inoculations and *Neopestalotiopsis* sp. (Pe_50_1) was the most aggressive in leaf inoculation assays. However, there were no significant differences in aggressiveness among the isolates in crown inoculation tests. This study corrects previous misidentification of *N. clavispora* in Spain, *confirming N. rosae* as the predominant species affecting the Spanish strawberry industry. These findings represent the first report of *Neopestalotiopsis* sp. and *N. iranensis* causing leaf spot and crown rot in Spain, providing essential molecular tools for accurate pathogen diagnosis and targeted disease management strategies.

## Introduction

Global strawberry (*Fragaria* x *ananassa* Duch.) production is about 10,728,811 tons across 436,067 hectares, with Europe contributing 1,194,930 tons from 75,025 hectares (FAOSTAT, 2024). Among European producers, Spain is a major producer with production of 323,540 tons from 7000 hectares (FAOSTAT, 2024). Geographically, strawberry cultivation in Spain is primarily concentrated in the Southwest region, particularly in Huelva province, which is known for its optimal agroclimatic conditions and sandy, slightly acidic soils that are ideal for strawberry (Medina, 2008). In addition to producing and exporting strawberry fruit, Spain also plays a key role as a major producer and exporter of strawberry transplants, which are cultivated in high-altitude nurseries in Castilla y León (Avilés et al., 2024; Medina, 2008).

During the last decade, one of the main problems in worldwide strawberry production has been the emergence of new fungal diseases and the resurgence of existing ones. These issues have been associated with strict regulations regarding the products used for soil disinfestation, frequent changes in cultivars, and the increasing exchange of planting materials, which facilitate the dispersion of plant pathogens (Avilés et al., 2024). This has been the case with *Phytophthora cactorum* (Lebert and Cohn) J. Schröt (Baggio et al., 2021a, Baggio et al., 2021b), *Macrophomina phaseolina* (Tassi) Goid, *Colletotrichum acutatum* J.H. Simmonds, *Fusarium oxysporum* f. sp. *fragariae* Winks and Y.N. Williams (Fof) (Avilés et al., 2008; Borrero et al., 2024; Pastrana et al., 2017; Prieto-Rodríguez et al., 2024; Marin et al., 2025) and *Neopestalotiopsis* spp (Avilés et al., 2024; Baggio et al., 2021a; Rebollar-Alviter et al., 2020).

Different *Pestalotiopsis*-like species, especially *Neopestalotiopsis*, have been reported in strawberry worldwide as causing fruit rot, leaf spot, root rot, and crown rot. *Pestalotiopsis*-like species primarily exhibit asexual (anamorphic) stages in their life cycle; however, a sexual stage has been documented in *Pinus*, *Quercus*, *Rhododendron*, and *Ricinus* spp., among others (Kobayashi et al., 2001; Maharachchikumbura et al., 2014; Shoemaker and Simpson, 1981). In the case of *Neopestalotiopsis*, conidia are the primary infective propagules (Espinoza et al., 2008). Species in this genus can produce a high number of conidia, which are dispersed in the air or by water splash (Prasannath et al., 2021). Potential sources of inoculum can be contaminated transplants from nurseries (McQuilken et al., 2004), diseased plant materials (Maharachchikumbura et al., 2011), and over-season strawberry crop debris, as described in Florida (Zuniga et al., 2024). Many species have been isolated as endophytes, but some species can remain quiescent until the plant is stressed and then switch to a pathogenic and necrotrophic lifestyle (Dardani et al., 2025; Liao et al., 2025; Maharachchikumbura et al., 2011). This lifestyle plasticity has been increasingly recognized as a critical factor in the emergence of aggressive *Neopestalotiopsis* strains in strawberry production systems worldwide, particularly following the widespread outbreaks first documented in Florida in 2018-2019 (Rebello et al., 2023; Ávila-Hernández et al., 2025).

The taxonomy of *Pestalotiopsis-*like species has been confusing since it has undergone multiple reclassifications over the years. Molecular analyses based on the internal transcribed spacer (ITS), translation elongation factor 1-alpha (TEF), and β-tubulin (TUB) gene regions, along with morphological characteristics, have been used to differentiate species and clarify phylogenetic relationships. This has led to the reclassification of *Pestalotiopsis*-like fungi into three genera: *Neopestalotiopsis*, *Pestalotiopsis*, and *Pseudopestalotiopsis* (Maharachchikumbura et al., 2014). The first report of *Pestalotiopsis-*like fungi causing strawberry fruit rot dates to 1972, when *Pestalotiopsis longisetula* (*Pestalotia longisetula)* was identified (Howard and Albregts, 1973). More recently, most isolates associated with strawberry foliar, root, and crown rot diseases have been reclassified as belonging to the genus *Neopestalotiopsis* (Baggio et al., 2021a; Chamorro et al., 2016; Obregon et al., 2018).

In Mexico, the causal agent of foliar, root and crown rot disease was identified as *Neopestalotiopsis rosae* (Rebollar-Alviter et al., 2020). In contrast, isolates from Florida included both *N. rosae* and a novel phylogenetic group classified as a cryptic *Neopestalotiopsis* sp., based on analyses of ITS, TEF, and TUB genes (Baggio et al., 2021a). In Florida, *N. rosae* was primarily found as a secondary pathogen on stressed plants, causing crown and root rot, leading to difficulties in plant establishment, although it could also be isolated from leaves and fruit. However, the more aggressive symptoms on leaves and fruit were mostly associated with the cryptic *Neopestalotiopsis* sp (Baggio et al., 2021a). Due to the unresolved taxonomy of *Neopestalotiopsis*, the term *Neopestalotiopsis* sp. will be used throughout the manuscript to refer to the cryptic species reported in Florida, strain 17-43 (Baggio et al., 2021a). Beyond Mexico and Florida, *Neopestalotiopsis* species such as *N. rosae*, *N. iranensis*, *N. hispanica* (syn. *N. vaccinii*), and *N. scalabiensis*, have been reported causing similar symptoms in other strawberry-producing countries, including Israel (Kenneth et al., 1968), Brazil (Camili et al., 2002), Egypt (Embaby, 2007), Morocco (Mouden et al., 2014), Iran (Ayoubi and Soleimani, 2016), China (Zhao, 2016), India (Mahapatra et al., 2018), Taiwan (Hsu et al., 2022), Bangladesh (Sultana et al., 2022), Italy (Dardani et al., 2025), and other states in the U.S. such as Indiana (Guan et al., 2023) and Ohio (Rotondo et al., 2023).

**Figure 1:**
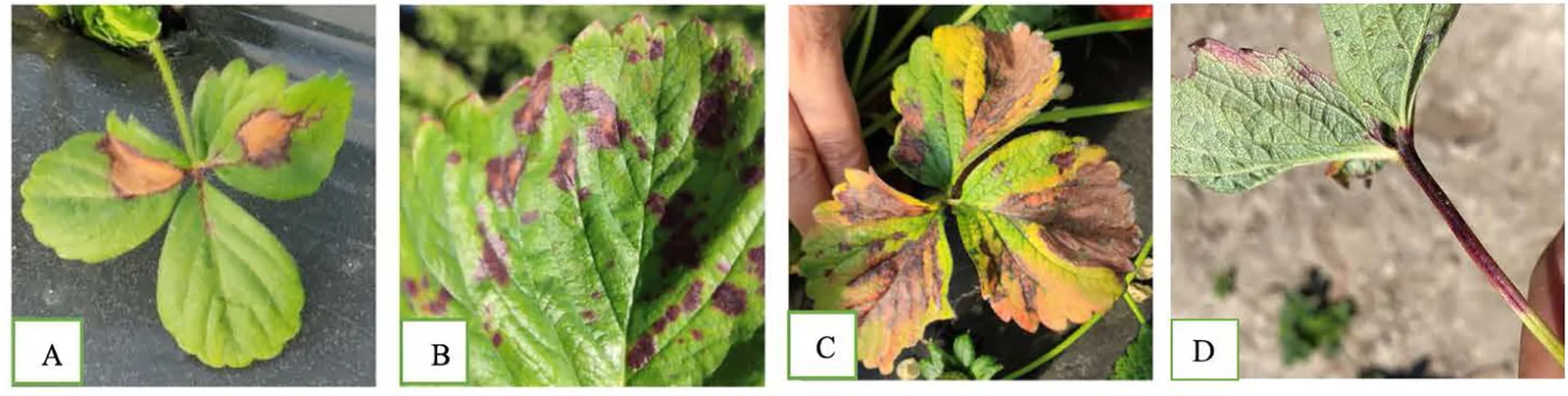
Symptoms of disease caused by *Neopestalotiopsis* spp. on strawberry plants in fruit production fields and nurseries in Spain. **(A)** Large light brown leaf spots developing from the center of the leaves; **(B)** Small light to dark brown and purple leaf spots; **(C)** Larger light to dark brown leaf spots developing from leaf tips or centerscenter; **(D)** Dark purple lesion on petiole.

In Spain, *Pestalotiopsis*-like fungi were initially associated with strawberry crown and root rot and identified as *N. clavispora* (Chamorro et al., 2016); however, they were later reclassified as *N. rosae* (Baggio et al., 2021a). In the same region in Spain, *N. clavispora* was also found affecting blueberries (Borrero et al., 2018). Before the 2019–2020 strawberry season in Spain, *Pestalotiopsis*-like fungi were isolated along with other root and crown pathogens, such as *Phytophthora cactorum* and *Colletotrichum acutatum*, and were considered opportunistic and secondary pathogens. However, starting in the 2020–21 strawberry season, these fungi were increasingly isolated from necrotic areas in the roots and crowns of weak and stunted transplants. More recently, the fungus has been associated with purple and light to dark brown leaf spots of varying sizes in certain cultivars (**Figures 1A–C**). Additionally, P*estalotiopsis*-like fungi were isolated from dark purple spots on petioles (**Figure 1D**). Under favorable conditions for disease development, particularly when nursery transplants are already quiescently infected, plants exhibit stunted growth, reduced leaf size, and overall diminished development, eventually dying three to five weeks after transplanting. These fungi can also cause significant plant establishment difficulties with great potential to negatively affect yield (Avilés et al., 2024).

We hypothesize that multiple genetically distinct *Pestalotiopsis*-like species are associated with strawberry diseases in Spain, and that their genetic diversity underlies differences in pathogenic potential and epidemiological relevance. Therefore, the main objective of this study are to: (i) characterize genetic variability among isolates using multilocus phylogenetic analyses and sequence-based molecular markers; (ii) evaluate morphology, pathogenicity and aggressiveness of representative isolates on strawberry plants using different cultivars; and (iii) integrate molecular and biological data to clarify species boundaries and assess their potential impact on the Spanish strawberry industry.

## Materials and methods

### Plant sampling and assessment

Symptomatic strawberry plants showing leaf spots, stunted growth or wilting were mainly collected between 2021 and 2023 from strawberry fruit production fields at Huelva and from nurseries in Granada and Castilla y León (Spain). In addition, this study included eight laboratory-preserved isolates stored in 50% glycerol at -80°C, previously recovered from strawberry, blueberry, and barley. Isolates were revived on potato dextrose agar (Condalab S. A., Torrejón de Ardoz, Madrid, Spain) supplemented with streptomycin (Merck Life Science S.L.U., Madrid, Spain) (100 mg/L) (PDA+S). In most cases, plants showed purple/brown leaf spots (**Figure 1**), wilting, and stunting. To recover fungi from plants, crown sections (0.5 cm) were rinsed with 0.1% Tween 20 (Sigma-Aldrich, Inc., St. Louis, MO, USA), submersed in 70% ethanol for 20 s, and in 1% NaOCl for 60 s. Later, crown sections were dried in a laminar flow hood (Azbil Telstar Technologies, S. L. U., Terrassa, Barcelona, Spain), placed on PDA+S, and incubated at room temperature (24 C and 10/14 h day/night). Colonies identified as *Pestalotiopsis*-like due to acervuli and appendage-bearing typical conidia production (Maharachchikumbura et al., 2014) were transferred to a new PDA+S plate. Single-conidium isolates were obtained by preparing a spore suspension from excised acervuli dispersed in sterile water agar (1 g/L). Serial dilutions were performed, and several drops from each dilution were plated onto fresh PDA+S plates. Plates were examined under a microscope to locate isolated conidia, which were individually marked and transferred to new PDA+S plates. After 20–24 h, germination was confirmed, and single-conidium cultures were incubated at room temperature until conidiomata developed. Cultures were then preserved on filter paper at –20 °C and in 50% glycerol at –80 °C (Sinclair and Dhingra, 1995).

### DNA extraction

DNA was extracted from a single-conidium isolate. In most cases, no differences in colony morphology were observed among single-conidium cultures grown on plates. However, when single-conidium cultures from the same original colony displayed distinct morphologies, separate DNA extractions were performed for each morphological type. Extractions were carried out using the HigherPurity™ Plant DNA Purification Kit (Canvax Reagents, S.L., Valladolid, Spain) following the manufacturer’s instructions with a modification: a small amount of fungal mycelium (50–100 mg) was scraped from a 7–14 days-old PDA+S plate and placed into a centrifuge tube containing lysis buffer and three to five glass beads. Subsequently, mycelium was homogenized by vortexing in 3 cycles of 7 m/s for 1 minute followed by 30-second rest intervals using a BeadBlaster™ 24 (Benchmark Scientific, Inc, NJ, USA). Genomic DNA was quantified using the Qubit 1x dsDNA HS Assay kit and a Qubit 4 fluorometer (Thermo Fisher Scientific, Life Technologies, Singapore) and adjusted to a final concentration of 5–25 ng/μL. The genomic DNA was then stored at −20 C for future use.

### DNA amplification and sequencing

Polymerase chain reaction (PCR) was used to amplify the partial internal transcribed spacer (ITS) using primers ITS5 (GGAAGTAAAAGTCGTAACAAGG) and ITS4 (TCCTCCGCTTATTGATATGC) (White et al., 1990), the *β*-*tubulin* (TUB) using primers T1 (AACATGCGTGAGATTGTAAGT) (O'Donnell and Cigelnik, 1997) and Bt-2b (ACCCTCAGTGTAGTGACCCTTGGC) (Glass and Donaldson, 1995), and the translation elongation factor 1-α (TEF) using primers EF1-526F (GTCGTYGTYATYGGHCAYGT) and EF1-1567R (ACHGTRCCRATACCACCRATCTT) (Rehner, 2001). PCR reaction contained molecular grade water, 1× Q5 Reaction Buffer (New England Biolabs, Inc., Ipswich, MA, USA), 200 µM dNTPs, 0.2 U/µL Q5 High-Fidelity DNA Polymerase (New England Biolabs, Inc., Ipswich, MA, USA), 5–25 ng DNA, and 0.5 µM (ITS), 0.4 µM (TUB) or 0.3 µM (TEF) for each primer. Amplifications were carried out in a T100 thermal cycler (Bio-Rad Laboratories, Inc., CA, USA) at 98 C for 30 s, 34 cycles of 10 s at 98 C, 30 s at 55°C, 56°C or 58°C (for ITS, TUB, or TEF, respectively), and 30 s at 72 C, followed by a final extension of 2 min at 72°C. Negative controls, in which sterile nuclease-free water was added to the reaction mixture instead of a DNA template, were included in the assays.

PCR products stained with RedSafe™ (Intro Biotechnology, South Korea) underwent electrophoresis in 1.5% agarose gels run in 1× TAE buffer at 120 V for 25 min and visualized over a UV transilluminator. PCR products were purified and sequenced by StabVida (Caparica, Portugal) using Sanger sequencing.

### Phylogenetics analysis

Before phylogenetic analysis, the sequences from the different genes were concatenated to a single alignment using MEGA11: Molecular Evolutionary Genetics Analysis version 11 (Tamura et al., 2021). The reference taxa employed in the phylogenetic analyses were retrieved from GenBank (**Table 1**). Phylogenetic assumptions, using the concatenated alignment, stationarity (constant nucleotide frequencies over time), and homogeneity (constant substitution rates over time) were assessed using matched-pairs tests of symmetry implemented in IQ-TREE (version 2.1.2; Minh et al., 2020; Naser-Khdour et al., 2019). The best-fit substitution model was then determined by ModelFinder considering gene partition according to the Bayesian information criterion (BIC) (Kalyaanamoorthy et al., 2017). For phylogenetic tree construction, partitioned Bayesian inference was performed using MrBayes (version 3.2.7; Ronquist et al., 2012) with the following settings: two independent runs, one million generations, sampling every 1,000 generations, and discarding the first 25% of trees as burn-in. The remaining trees were used for generating a 50% majority-rule consensus tree with posterior probabilities for each node. In addition, maximum likelihood analysis was conducted using IQ-TREE with the same partition scheme and best-fit models. Branch support was estimated based on 1,000 standard bootstrap replicates (Chernomor et al., 2016) and Shimodaira-Hasegawa–like approximate likelihood ratio test (SH-aLRT) (Guindon et al., 2010). The phylogenetic trees were rooted to *Pestalotiopsis trachicarpicola* (OP068) and visualized and edited using FigTree (version 1.4.4; http://tree.bio.ed.ac.uk/software/figtree/) and Inkscape (version 1.0.1; https://inkscape.org/), respectively. After analysis of the first 143 isolates, only the TUB gene was sequenced for the subsequent isolates. All sequences have been deposited in GenBank (**Supplementary Table 1**).

**Table 1:** Details of the reference species used for sequence comparisons and phylogenetic analysis.

Species^r^	Accession^s^	Host^t^	Location^u^	ITS^v^	TEF^w^	TUB^x^	Reference
*N. rosae*	PEST3	*Fragaria x ananassa*	Egypt	KY688075	KY688074	KY688073	Essa et al. (2018)
*N. rosae*	7927	*Fragaria x ananassa*	Australia	KY271740	KY271093	KY271094	N/A
*N. rosae*	CBS 124745	*Paeonia suffruticosa*	USA	KM199360	KM199524	KM199430	Maharachchikumbura et al. (2014)
*N. rosae*	ASM2307868	N/A	Taiwan	JALGAS000000000^z^			Hsu et al. (2022)
*N. rosae*	CBS 101057	*Rosa* sp.	New Zealand	KM199359	KM199523	KM199429	Maharachchikumbura et al. (2014)
*N. rosae*	CRMFRC	*Fragaria x ananassa*	Mexico	MN385718	MN268532	MN268529	Rebollar-Alviter et al. (2020)
*N. rosae*	CRMFRH	*Fragaria x ananassa*	Mexico	MN385719	MN268533	MN268530	Rebollar-Alviter et al. (2020)
*N. rosae ^y^*	TOR-802-803-804	*Fragaria x ananassa*	Spain	KU096879	KU096881	KU096880	Chamorro et al. (2016)
*N. rosae* 13481 ^z^	N/A	*Fragaria x ananassa*	USA	N/A	N/A	N/A	Han et al., 2024
*N.* sp. 1902 ^z^	N/A	*Fragaria x ananassa*	USA	N/A	N/A	N/A	Han et al., 2024
*N. iranensis*	CBS 137768	*Fragaria x ananassa*	Iran	KM074048	KM074051	KM074057	Ayoubi and Soleimani (2016)
*N. mesopotamica*	CBS29974	*Eucalyptus* sp.	Turkey	KM199361	KM199541	KM199435	Maharachchikumbura et al. (2014)
*N. cubana*	CBS60096	Leaf litter	Cuba	KM199347	KM199521	KM199438	Maharachchikumbura et al. (2014)
*N. saprophytica*	CBS115452	*Litsea rotundifolia*	Hong Kong	KM199345	KM199538	KM199433	Maharachchikumbura et al. (2014)
*N. aotearoa*	CBS36754	*Canvas*	New Zealand	KM199369	KM199526	KM199454	Maharachchikumbura et al. (2014)
*N. piceana*	CBS25432	*Cocos nucifera*	Indonesia	KM199372	KM199529	KM199452	Maharachchikumbura et al. (2014)
*N. ellipsospora*	CBS115113	*Ardisia crenata*	Hong Kong	KM199343	KM199544	KM199450	Maharachchikumbura et al. (2014)
*N. samarangensis*	CBS115451	*Unidentified tree*	China	KM199365	KM199556	KM199447	Maharachchikumbura et al. (2014)
*N. formicarum*	CBS11583	*Plant debris*	Cuba	KM199344	KM199519	KM199444	Maharachchikumbura et al. (2014)
*N. honoluluana*	CBS111535	*Telopea* sp.	USA	KM199363	KM199546	KM199461	Maharachchikumbura et al. (2014)
*N. natalensis*	CBS13841	*Acacia mollissima*	South Africa	KM199377	KM199552	KM199466	Maharachchikumbura et al. (2014)
*N. protearum*	CBS114178	*Leucospermum cuneiforme* cv.	Zimbabwe	JN712498	KM199542	KM199463	Maharachchikumbura et al. (2014)
*N. clavispora*	MFLUCC120280	*Magnolia* sp.	China	JX398978	JX399044	JX399013	Maharachchikumbura et al. (2012)
*P.trachicarpicola*	OP068	*Trachycarpus fortunei*	China	JQ845947	JQ845946	JQ845945	Maharachchikumbura et al. (2014)

^r^ Neopestalotiopsis (N.) or Pestalotiopsis (P.) species.

^s^ Official name in the culture collection from which the isolate originated.

^t^ Host or tissue from which the isolate was recovered.

^u^ Country where the isolate was collected.

^v^ GenBank accession number for the internal transcribed spacer (ITS) region.

^w^ GenBank accession number for the translation elongation factor (TEF) region.

^x^ GenBank accession number for the -β-tubulin (TUB) region.

^y^ Isolate from Spain was first identified as Neopestalotiopsis clavispora (Chamorro et al., 2016) and later re-identified as N. rosae by Baggio et al. (2021a). In this study, we confirmed its identity as N. rosae.

^z^ Sequences were extracted from the whole genome sequence.

N/A, reference not available.

**Figure 2:**
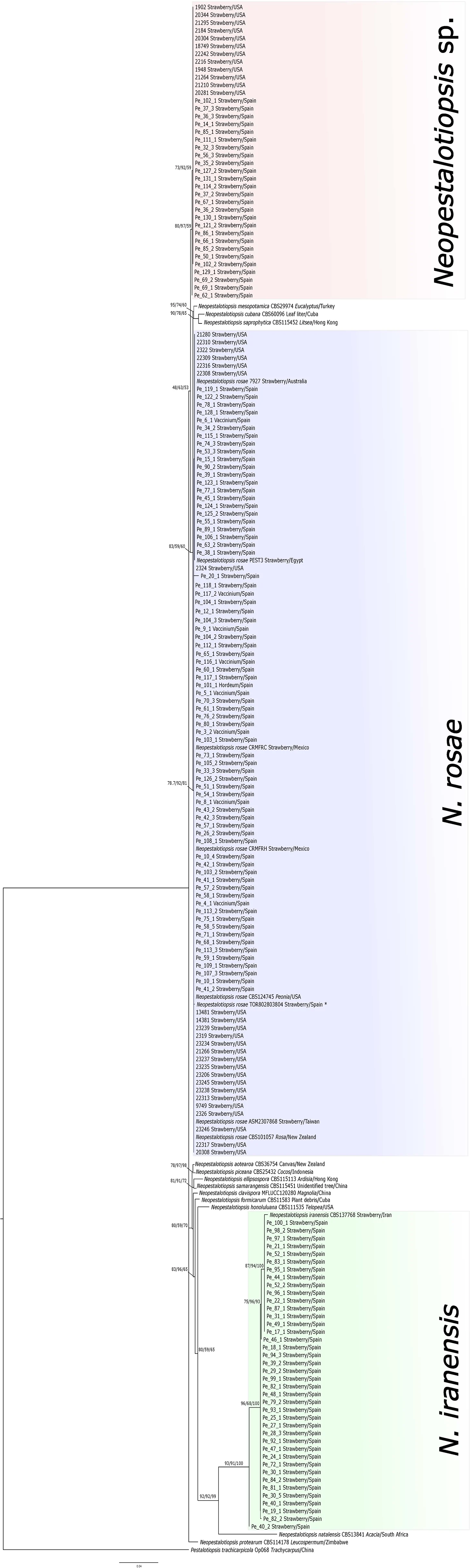
Maximum likelihood phylogenetic tree based on concatenated sequences of three loci [internal transcribed spacer (ITS), elongation factor (TEF), and β-tubulin (TUB)] for *Pestalotiopsis*-like isolates. The Shimodaira-Hasegawa-like approximate likelihood ratio test bootstrap support values (1,000 replicates), and ultrafast bootstrap support values (1,000 replicates) are given at the nodes. The dataset includes 143 isolates and reference sequences retrieved from GenBank (**Table 1**).

In isolates collected up until the 2021–2022 strawberry season (n = 143), phylogenetic analyses were initially performed using individual gene regions (data not shown). Subsequently, a phylogenetic tree was constructed based on concatenated gene sequences (**Figure 2**). The divergence between ITS and TEF sequences was insufficient to differentiate species. Notably, the phylogenetic tree generated using TUB sequences was similar to that obtained with concatenated sequences (**Figure 2**). Therefore, for isolates collected from the 2022–2023 strawberry season onward (n=92), phylogenetic analysis was conducted exclusively using TUB sequences (**Supplementary Figure 1**).

### Morphological characterization

Morphological descriptions were made for six isolates, two *N. rosae* (Pe_26_2 and Pe_78_1), two *Neopestalotiopsis* sp. (Pe_50_1 and Pe_129_1), and two *N. iranensis* (Pe_47_1 and Pe_97_1) grown in PDA (20%) at room temperature (~25°C) under daylight. These isolates were chosen randomly to maximize diversity, as each was recovered from a different strawberry cultivar and from different locations across Spain. Autoclaved pine needles were placed on synthetic nutrient-poor agar (PNA) (Crous et al., 2009) to observe conidiomatal development. Microscopic preparations were made in distilled water. For each isolate, 100 images showing multiple conidia were captured; however, not all conidia were suitable for measuring all morphological structures. Therefore, 30 measurements per structure were obtained from conidia in which the relevant features were clearly visible. Observations were performed using a Leica DM500 microscope coupled to a camera Leica ICC50 W with Leica Application Suite version 3.4.0. software (Leica Microsystems, Wetzlar, Germany). The same isolates used for morphological characterization were selected for the pathogenicity assays to ensure consistency across analyses.

### Pathogenicity assays

#### Experimental design and growth conditions

Pathogenicity was evaluated using a randomized block design with five replicates. An experimental unit consisted of a 10 × 10 × 11 cm plastic pot (Pöppelmann TEKU^®^, Lohne, Germany) filled with a 1:1 sand-to-peat substrate mix (Kekkilä Professional, Projar, S.L., Quart de Poblet, Valencia, Spain), supplemented with 4 g/L of Osmocote 15-9-12 + 2MgO+TE (with nutrient release duration of 5–6 months) (ICL Specialty Fertilizers Iberia, S.L., Murcia, Spain). Each pot contained one bare-root strawberry transplant with at least three to four fully developed leaves. Six isolates were used in all assays: two *N. rosae* (Pe_26_2 and Pe_78_1), two *Neopestalotiopsis* sp. (Pe_50_1 and Pe_129_1), and two *N. iranensis* (Pe_47_1 and Pe_97_1). These isolates were inoculated onto strawberry cultivars ‘Rociera FNM’, ‘Palmeritas’, and ‘Plared 15105’. Each assay included the factors: isolate (six levels), cultivar (three levels), and block (five levels), resulting in 90 experimental units (6 isolates x 3 cultivars x 5 blocks). A negative control receiving only sterile distilled water was included in each assay. Negative control plants remained symptomless and were not included in the statistical analysis.

Four independent assays were conducted: two focused on foliar inoculation (F1, F2) and two on crown inoculation (C1, C2). The experiments were conducted in a glass greenhouse at the Escuela Técnica Superior de Ingeniería Agronómica (E.T.S.I.A.) field research station, University of Seville (37°21′09″N, 5°56′11″W) from June to September 2023. The greenhouse cooling system was activated at 30 C, with a fogger triggered at 32 C. A shading net was deployed from 9 AM to 5 PM. Before inoculation, plants were overhead irrigated for 2 minutes every 2 hours throughout daylight hours to support establishment. After inoculation, experimental units were covered with transparent plastic bags for two days to maintain humidity. Subsequently, plants were irrigated via a drip system twelve times daily, with each cycle lasting two minutes, until the experiment concluded.

#### Inoculum production

*Neopestalotiopsis* isolates were grown on PDA+S at room temperature (25 C) for seven to ten days. All 1-cm-diameter plugs from a fully colonized 90-mm Petri plate were evenly distributed into two sterile rice bags (330 g rice with 240 ml of water per bag). White rice was first autoclaved in polypropylene bags with filters (PPD75/REH/V37-53, SacO2, Deinze, Belgium) according to the procedure described by Benson and Parker (2016). The inoculated rice bags were closed and incubated at room temperature (25 C) for 20 days. Once the fungus had completely colonized the rice bags (15 to 20 days), the contents were added to a one-liter sterile plastic bottle containing 0.01% water agar and ten sterile marbles. Subsequently, the mixture was shaken in the orbital shaker (Orbitron, Infors HT, Bottmingen, Switzerland) at 200 rpm for one hour. The resulting suspension was filtered through three layers of sterile cheesecloth. It was then left to settle for two hours to allow conidia to precipitate at the bottom. The supernatant was carefully removed using a pipette, conidia were counted with a hemocytometer, and the final inoculum was adjusted to 10^6^; conidia/mL with sterile distilled water.

#### Inoculation

*Crown inoculation*. Plants were wounded at the base of the crown (Rebollar-Alviter et al., 2020) with four incisions using a sterile 0.5 mm diameter needle. Immediately after wounding, 10 mL of a 10^6^ conidia/mL suspension was applied to the incised area using a pipette. For negative control plants, 10 mL of sterile water was used instead. The crown was then covered with substrate.

*Leaf inoculation*. Once plants were established and had developed at least three to four leaves, they were hand-pump-sprayed with 2 mL of a 10^6^ conidia/mL suspension of the corresponding isolate. Negative control plants received 2 mL of sterile water.

#### Disease scoring and confirmation of Koch’s postulates

The incidence of the disease (proportion of diseased plants out of the total across the five replicates of each isolate × cultivar combination in each trial) and severity were assessed 40 days post-inoculation in all assays. In crown inoculation assays, severity was calculated as the proportion of symptomatic leaves per plant, where leaves showing chlorosis, wilting, or necrosis were considered symptomatic. In cases where plants were severely affected (e.g., stunted or dead), all leaves were considered symptomatic. In foliar assays, it was recorded as the percentage of necrotic leaf area per plant.

To fulfil Koch’s postulates, the causal agent was re-isolated from the crowns and roots of symptomatic plants onto PDA+S medium, thereby confirming the presence of *Neopestalotiopsis*-like infection. Crowns were cut longitudinally in half, and four pieces of approximately 0.5 cm^2^ were removed from each half, including both necrotic and adjacent non-necrotic tissue. In addition, four segments of approximately 1 to 2 cm taken from roots were assayed from each symptomatic plant. Crown and root pieces were rinsed with 0.1% Tween 20, followed by immersion in 70% ethanol for 20 s and 1% sodium hypochlorite for 60 s. When all tissue samples were dried, they were added onto plates with PDA+S. After incubation, pathogen identification was confirmed based on morphological characteristics.

### Statistical analysis

The morphometric data of the conidia were analyzed using one-way analysis of variance (ANOVA) followed by LSD tests (P < 0.05). For the pathogenicity assays, the dependent variables, incidence and severity, were recorded forty days after inoculation, both in the crown and leaf inoculations. The data from the two repetitions of assay with the same type of inoculation for both dependent variables were combined after checking that there were no significant differences among them (P < 0.05). Consequently, a factorial ANOVA was performed where the isolate and cultivar were treated as fixed effects, while the assay and blocks nested within the assay were considered random effects. For the dependent variable ‘incidence’, the factors included were: isolate, cultivar, assay, and the interaction between isolate × cultivar. For the dependent variable ‘severity’, the factors were: isolate, cultivar, assay, nested block in assay, and the interaction between isolate × cultivar. Mean comparisons were performed using Duncan’s multiple range test (P < 0.05). In all cases, before ANOVA, the data were checked for normality (Shapiro–Wilk) and homoscedasticity (Levene’s test) and transformations were applied when necessary (shown in **Table 2**). All statistical analyses were performed using Statgraphics Centurion 18 (version 18.1.13, Statgraphics Technologies, Inc., The Plains, VA, USA).

**Table 2:** Disease incidence and severity on strawberry cultivars ‘Plared 15105’, Rociera FNM’, and ‘Palmeritas’ inoculated with *Neopestalotiopsis rosae*, *Neopestalotiopsis* sp. and *Neopestalotiopsis iranensis* isolates.

	Foliar inoculation		Crown inoculation	
Isolates	Incidence	Severity	Incidence	Severity
*Neopestalotiopsis iranensis* (Pe_47_1)	0.13 c ^y^	0.01 c ^z^	0.37^ns^	0.14^ns^
*Neopestalotiopsis rosae* (Pe_78_1)	0.30 bc	0.03 bc	0.43	0.18
*Neopestalotiopsis* sp. (Pe_129_1)	0.30 bc	0.04 bc	0.20	0.11
*Neopestalotiopsis iranensis (*Pe_97_1)	0.40 bc	0.04 bc	0.50	0.25
*Neopestalotiopsis rosae* (Pe_26_2)	0.43 ab	0.05 b	0.37	0.13
*Neopestalotiopsis* sp. (Pe_50_1)	0.67 a	0.11 a	0.33	0.18
Cultivars				
‘Plared 15105’	0.18 b	0.03 b	0.27 b	0.13^ns^
‘Rociera FNM’	0.30 b	0.05 a	0.34 ab	0.13
‘Palmeritas’	0.63 a	0.06 a	0.50 a	0.23

^y^ Mean disease incidence (n=6 for isolate and n=12 for cultivar) and severity (n=30 for isolate and n=60 for cultivar) in the same column followed by different letters are significantly different according to Duncan´s significant difference test at *P* < 0.05.

^z^ To satisfy the assumptions of normality and homogeneity of variance, the data on severity following leaf inoculation were transformed using the square root, whilst the data on severity following crown inoculation were transformed using the logarithm prior to statistical analysis.

ns, no significant statistical effect (*P* < 0.05) of isolates or cultivars.

## Results

### Phylogenetic analysis

Phylogenetic analysis based on concatenated sequences of TEF, ITS, and TUB genes of isolates recovered up to the 2021–2022 strawberry season revealed that sixty-two *Neopestalotiopsis* isolates from strawberry plants, eleven isolates from blueberry plants, and one isolate from barley clustered within the same clade as *N. rosae*. Twenty-six *Neopestalotiopsis* isolates from strawberry plants clustered within the cryptic *Neopestalotiopsis* sp. clade previously reported in Florida (Baggio et al., 2021a). Additional phylogenetic analyses, including *N. hispanica* reference strains, indicated that isolate 17–43 clustered closely with *N. hispanica*; however, due to limited resolution, it was retained as *Neopestalotiopsis* sp. in this study. The remaining forty Spanish *Neopestalotiopsis* isolates clustered within the same clade as *N. iranensis* CBS137768, originally recovered from symptomatic strawberry plants in Iran (**Figure 2**) (Ayoubi and Soleimani, 2016). A previously reported strawberry isolate from Spain, initially classified as *N. clavispora* TOR-802-803-804 (Chamorro et al., 2016) and later reclassified as *N. rosae* (Baggio et al., 2021a), also clustered in our study with *N. rosae*. The Pe_9 and Pe_10 isolates that were previously reported as *N. clavispora* in blueberry in Spain (Borrero et al., 2018) have also been reclassified in this study as *N. rosae*. No Spanish *Neopestalotiopsis* isolates from this study clustered within the *N. clavispora* clade (**Figure 2**).

Phylogenetic analysis based on the TUB gene of isolates recovered during the 2022–2023 strawberry season revealed that only five of the ninety-two strawberry isolates clustered within *N. iranensis* clade. In contrast, sixty-three isolates from the same season clustered with *N. rosae*, whereas twenty-four isolates were placed in the *Neopestalotiopsis* sp. clade (**Supplementary Figure 1**).

#### Morphological characterization

Fungal colonies were white, and cottony on the upper surface, regardless of the isolate. However, on the reverse side, isolates from the *Neopestalotiopsis* sp. and *N. iranensis* were white to pale yellow, whereas those of *N. rosae* were pale luteous to orange (**Supplementary Figure 2**). Conidiomata exuding black conidial masses were gregarious in *N. iranensis* and concentric in *N. rosae* and *Neopestalotiopsis* sp. Spores of all isolates were ellipsoid to fusiform, five-celled, and versicolored, with three median cells colored. Basal and apical cells were hyaline, whereas the second cell from the base was light brown, the third cell was honey brown, and the fourth cell was brown. Spores had one basal and several apical appendages, the number of apical appendages ranged from three to five (mostly four) in *N. iranensis* conidia and from two to four (mostly three) in *N. rosae* and *Neopestalotiopsis* sp. (**Supplementary Figure 3**).

Spore length was higher in *N. iranensis* isolates than in *Neopestalotiopsis* sp., while *N. rosae* showed intermediate values, not differing significantly from either group (*P < 0.05*). Spore length ranged from 27.4 to 33.1 μm (mean = 27.4 μm) for *N. iranensis*, 21.4 to 31.6 μm (mean = 26.7 μm) for *N. rosae*, and 18.8 to 32.7 μm (mean = 26.2 μm) for *Neopestalotiopsis* sp. Spore width was significantly higher in *N. iranensis* compared to the other groups (*P < 0.0001*), ranging from 4.9 to 8.5 μm (mean = 7.0 μm), while *N. rosae* ranged from 5.7 to 10.0 μm (mean = 7.4 μm) and *Neopestalotiopsis* sp. from 5.6 to 9.6 μm (mean = 7.7 μm). Apical appendages were significantly longer in *N. iranensis* isolates than in the other two species (*P < 0.0001*). They ranged from 31.4 to 61.6 μm (mean = 45.5 μm) in *N. iranensis*, 23.4 to 46.0 μm (mean = 33.8 μm) in *N. rosae*, and 22.5 to 54.9 μm (mean = 35.1 μm) in *Neopestalotiopsis* sp. In contrast, basal appendages were shorter in *Neopestalotiopsis* sp. compared to the other species (*P < 0.01*). Values ranged from 5.0 to 16.7 μm (mean = 10.5 μm) in *N. iranensis*, 5.7 to 18.8 μm (mean = 10.0 μm) in *N. rosae*, and 3.7 to 15.0 μm (mean = 8.6 μm) in *Neopestalotiopsis* sp.

### Pathogenicity tests

Non-inoculated control plants remained healthy in all assays and were not included in the statistical analysis. In the factorial ANOVA for the incidence of crown and leaf inoculations, the factors included were: isolate, cultivar, assay, and the interaction between isolate × cultivar, with only isolate and cultivar being significant (**Supplementary Table 2**). In the factorial ANOVA for severity of crown and leaf inoculations, the factors were: isolate, cultivar, assay, block nested in the assay, and the interaction between isolate × cultivar, and only isolate and cultivar were significant (**Supplementary Table 3**). Therefore, we present the effects of these two main factors on both dependent variables, after the corresponding test of separation of means (**Table 2**).

*Foliar inoculation*. Forty days after inoculation, the six tested isolates produced leaf lesions on all cultivars (**Table 2**). However, *Neopestalotiopsis* sp. (Pe_50_1) caused the most disease severity and, together with *N. rosae* (Pe_26_2), had the highest disease incidence. *Neopestalotiopsis iranensis* (Pe_47_1) was the least aggressive, although it did not show significant differences form the isolates of *N. iranensis* (Pe_97_1), *N.* sp. (Pe_129_1), and *N. rosae* (Pe_78_1). All three cultivars developed symptoms across all isolates tested, though susceptibility varied among cultivars. Based on incidence data from foliar inoculation, ‘Palmeritas’ was the most susceptible cultivar compared to ‘Rociera FNM’ and ‘Plared 15105’. In contrast, severity data indicated that ‘Plared 15105’ was the least susceptible cultivar (**Table 2**).

*Crown inoculation.* Symptoms (wilted, chlorotic, stunted, or dead leaves or plants) were observed after forty days. When analyzing the recorded data from the plants of the three cultivars, the incidence of the disease recorded forty days after inoculation ranged from 20 to 50% and the severity from 11 to 25%, but without significant differences among the isolates (**Table 2**). In all cultivars and against all isolates, plants with symptoms such as leaf spots (large light brown leaf spots developing from the center of the leaves, small light to dark brown and purple leaf spots, or larger light to dark brown leaf spots developing from the tips or the center of the leaves), stunted growth, or death were observed. Based on disease incidence, the cultivars showed different levels of susceptibility. ‘Palmeritas’ was more susceptible than ‘Plared 15105’, and ‘Rociera FNM’ did not show a significant difference in susceptibility to the other cultivars (**Table 2**).

The morphological characteristics of isolates recovered from symptomatic plants showing necrotic leaf spots, crown, and root lesions, as well as their spore production, were consistent with those of the original inoculum, as described in the pathogenicity test subsection of the Materials and Methods section. Pestalotiopsis-like fungi were not recovered from any non-inoculated plants.

## Discussion

Historically, the first identifications of *Pestalotiopsis*-like pathogens responsible for strawberry fruit rot were reported by Howard and Albregts (1973), which were linked to *Pestalotia longisetula* (syn. *Pestalotiopsis longisetula*, Guba 1961; Index Fungorum, 2025). More recent studies have associated strawberry isolates with the genus *Neopestalotiopsis* (Baggio et al., 2021a; Chamorro et al., 2016; Erdurmuş et al., 2022; Gilardi et al., 2019; Lawrence et al., 2023; Machín et al., 2019; Morales-Mora et al., 2019; Obregon et al., 2018; Rebollar et al., 2020; Rotondo et al., 2023; Sun et al., 2021; Wu et al., 2020). In this study, we utilized phylogenetic analysis and morphology characterization to establish that diseases caused by *Pestalotiopsis*-like fungi on strawberry in Spain are also primarily associated with the genus *Neopestalotiopsis*. The strains and species included in our phylogenetic analysis were selected to follow consistent patterns established in previous studies, considering current nomenclature and taxonomic understanding (**Table 1**). Species that were too distantly related were excluded to maintain meaningful phylogenetic resolution and clarity in the tree. A phylogenetic analysis including *N. hispanica* (CBS 147686) and *N. iberica* (CBS 147688) was also conducted (**Supplementary Figure 4**). Although isolate 17–43 showed close affinity to *N. hispanica*, we considered that the available reference data do not provide sufficient resolution for confident species-level identification. The original descriptions of *N. hispanica* and *N. iberica* may not have fully adhered to current taxonomic standards, and the sequences available from type material are relatively short, which limits robust phylogenetic resolution. The International Commission on the Taxonomy of Fungi (ICTF) recommends the use of genealogical concordance analyses for delimiting cryptic species whenever feasible. This approach should include the publication of single-locus phylogenies (e.g., as **Supplementary Material**) alongside the concatenated multilocus phylogenetic tree (Aime et al., 2021). Moreover, individual gene trees based on ITS, TUB, and EF sequences do not support either of these species as monophyletic groups, further emphasizing the need for multilocus analyses in accurate species delimitation. Therefore, while our data indicate that strain 17–43 is closely related to *N. hispanica*, we have retained it as *Neopestalotiopsis* sp. in the manuscript to avoid potential misidentification.

Isolates recovered in this study, as well as those obtained in previous years, were separated into three distinct groups. The first group, comprising 52.5% of the recovered isolates was identified as *N. rosae*, which was also the only species previously identified in Mexico (Rebollar-Alviter et al., 2020). The second group (18.7%) clustered with a newly described cryptic *Neopestalotiopsis* sp (Baggio et al., 2021a). The third group (28.8%) comprised genetically distinct isolates belonging to *N. iranensis*. Although the majority of our isolates grouped consistently within the *N. rosae, N. iranensis*, and *Neopestalotiopsis* sp. clades, several recent studies have noted that these clades are not phylogenetically stable and may vary depending on the loci analyzed and the taxon sampling used. This instability highlights the cryptic nature of *Neopestalotiopsis* species boundaries and suggests that the relationships observed here should be interpreted with caution. Continued integration of multilocus and genomic data will be important to resolve species limits within this complex. The morphological characteristics of the *N. iranensis*, *N. rosae*, and *Neopestalotiopsis* sp. isolates in this study were also consistent with previously reported descriptions for these species (Ayoubi and Soleimani, 2016; Baggio et al., 2021a; Maharachchikumbura et al., 2014). In Spain, *N. clavispora* was previously identified as the species causing crown rot symptoms on strawberry (Chamorro et al., 2016) based on BLAST analysis. However, Baggio et al. (2021a) reclassified this isolate (TOR-802-803-804) as *N. rosae* using phylogenetic analysis, and our phylogenetic results are consistent with this reclassification, confirming that the isolate belongs to *N. rosae*. Furthermore, *N. clavispora* was distantly related to *N. rosae*, clustering in a separate clade. None of the Spanish isolates from our extensive surveys grouped within this clade, raising questions about the association of *N. clavispora* with strawberry in Spain.

As in previous reports, multiple genes were targeted to characterize the isolates in this study (Ayoubi and Soleimani, 2016; Baggio et al., 2021a; Erdurmuş et al., 2022; Essa et al., 2018; Lawrence et al., 2023; Rebollar-Alviter et al., 2020; Rotondo et al., 2023). However, our findings indicate that phylogenetic trees based solely on TUB sequences produce a topology similar to those constructed from concatenated TEF-ITS-TUB sequences in terms of separating *Neopestalotiopsis* sp. from *N. rosae* and *N. iranensis* in strawberry. Nonetheless, using a concatenated alignment of ITS, TEF, and TUB is recommended, as it provides improved species resolution and stronger clade support for the reference type species included in the phylogenetic analysis. Previous studies in Florida also found that TUB sequences were the most variable for distinguishing species associated with strawberry, while the divergence in ITS was too low to separate them (Baggio et al., 2021a; Rebello et al., 2023). Polymorphisms within the TUB region have been well characterized. Kumar et al. (2018) identified these variations among Florida isolates of *Neopestalotiopsis* sp., *N. rosae*, and other related species using multiple sequence alignment. Subsequently, Rebello et al. (2023) employed High-Resolution Melting (HRM) analysis targeting the TUB region, successfully distinguishing *Neopestalotiopsis* isolates from strawberry in Florida into two distinct groups: *Neopestalotiopsis* sp. and *N. rosae*. More recently, Han et al. (2024) performed chromosome-scale whole genome sequencing on two *Neopestalotiopsis* isolates representing two groups *Neopestalotiopsis* sp. (*19*-02), and *N. rosae* (13-481), and the TUB region emerged as a robust marker for distinguishing between groups. These findings collectively reinforce the utility of the TUB region as one of the most informative and reliable genetic markers for differentiating *Neopestalotiopsis* species recovered from strawberry.

Unlike the clear association between *Neopestalotiopsis* species and the plant tissues from which they were isolated in Florida (e.g., *N. rosae* predominantly from roots and crowns, and *Neopestalotiopsis* sp. from leaves and fruit) (Baggio et al., 2021a), our findings in Spain did not reveal a similar pattern of species distribution across strawberry tissues. Examining the strawberry tissues from which they were isolated, among the eleven isolates from leaves, most clustered within *N. rosae* or *Neopestalotiopsis* sp., with only one isolate identified as *N. iranensis*. Isolates from petioles were also distributed among the three species. Additionally, of the four isolates recovered from crowns that had remained in Huelva strawberry fields over the summer, two were identified as *N. rosae*, one as *Neopestalotiopsis* sp., and the fourth as *N. iranensis*. In Iran, *N. iranensis* has previously been isolated from rotted strawberry fruit, stolons, and leaf lesions (Ayoubi and Soleimani, 2016). However, in Spain, we did not recover any *Neopestalotiopsis* isolates from strawberry fruit. In contrast, HRM analysis of soil and crown strawberry debris under Florida summer conditions revealed that most of the *Neopestalotiopsis* colonies recovered belonged to the newly identified *Neopestalotiopsis* sp. clade (Zuniga et al., 2024). Future studies in Spain should include additional sampling and HRM analysis on Spanish soil and crown isolates surviving summer conditions to determine if *Neopestalotiopsis* sp. predominates in these environments or if a mixture of the three identified clades is present instead.

Among the 231 Spanish isolates characterized, when isolated from non-strawberry hosts, the species was always *N. rosae.* Specifically, all isolates obtained from blueberry and the sole isolate from barley were identified as *N. rosae*, including those previously classified as *N. clavispora*. However, given the limited number of isolates from non-strawberry hosts, further sampling is required to confirm this pattern. On the other hand, we have observed that there is a certain association between *Neopestalotiopsis* species and strawberry cultivars. Thus, *N. rosae* is mostly isolated from ‘Leticia’ and ‘Palmeritas’; *Neopestalotiopsis* sp. from ‘Leticia’; and *N. iranensis* is nearly equally recovered from ‘Calinda’, ‘Leticia’, and ‘Fortuna’.

Mixed infections involving more than one *Neopestalotiopsis* species were also detected within the same plant. For instance, isolates Pe_158_1 and Pe_156_1 were obtained from the same plant, with Pe_158_1 (from the root) identified as *N. rosae* and Pe_156_1 (from the crown) identified as *Neopestalotiopsis* sp. Furthermore, in one occurrence where DNA from multiple single-conidium isolates from the same source was extracted and sequenced (Pe_39), differentiation into two distinct species was observed: Pe_39_1 was identified as *N. rosae* and Pe_39_2 as *Neopestalotiopsis* sp. These findings highlight the possibility of co-infection by multiple *Neopestalotiopsis* species within a single plant and underscore the importance of working with single-conidium isolates for accurate species characterization.

Regarding the origin of the strawberry samples, isolates from strawberry production fields in Huelva were clustered into the three described groups, as were the isolates from nurseries in Castilla y León. However, the isolates from nurseries in Granada were only grouped within *N. rosae* and *Neopestalotiopsis* sp., with no recovery of *N. iranensis* from this nursery location. *Neopestalotiopsis* sp. outbreaks in both Florida and Canada were originally linked to a nursery in North Carolina that supplies plants to both locations (Baggio et al., 2021a; McNally et al., 2023). In México, the initial reports of *N. rosae* were associated with open-field nurseries in the state of Michoacán (Rebollar-Alviter et al., 2020). In Spain, *Neopestalotiopsis* sp. and *N. rosae* have been isolated from transplants originating from nurseries in both Castilla y León and Granada. Given that many Spanish nurseries also export plants to Italy, it is notable that no isolate of *Neopestalotiopsis* sp. was detected there in the recent study by Dardani et al. (2025). In Spain, the presence of *N. iranensis* isolates may originate from nursery transplants, but also result from soil or infected crowns that remained in non-rotated strawberry fields over the summer.

Pathogenicity tests using foliar inoculation showed that isolate Pe_50_1, representing *Neopestalotiopsis* sp., caused significantly higher disease severity compared to other isolates. Although the incidence was relatively high at 67%, the severity remained low at 11%. In contrast, another *Neopestalotiopsis* sp. isolate, Pe_129_1, caused only 30% incidence and 3% severity, with no statistically significant difference from the *N. iranensis* isolate Pe_47_1, which exhibited the lowest level of disease. These findings suggest that there may be a variation in host-pathogen interaction among *Neopestalotiopsis* sp. isolates because Florida isolates from this clade were reported as always being associated with severe outbreaks in the field (Rebello et al, 2023). However, crown inoculation did not reveal significant differences in aggressiveness among isolates. In recent Spanish strawberry seasons, foliar symptoms associated with *Neopestalotiopsis* have become increasingly prevalent and diverse. Nevertheless, in some cases, the fungus was difficult to isolate from symptomatic leaves, even after incubation in a high-humidity chamber or direct culturing. This suggests that these spots may not always result from direct fungal colonization. Instead, they could be secondary manifestations triggered by systemic infection from the crown or roots or be influenced by abiotic stress factors.

In terms of cultivar susceptibility, ‘Palmeritas’ exhibited the highest susceptibility after foliar inoculation, whereas ‘Plared 15105’ displayed the lowest susceptibility after crown inoculation. These pathogenicity trials were conducted under long-day and high-temperature conditions, favoring runner production in short-day cultivars such as ‘Palmeritas’ and ‘Rociera FMN’ but allowing day-neutral cultivars like ‘Plared 15105’ to develop multiple crowns (Lauricella, 2021). Post-inoculation analysis revealed that although the initially infected crowns in ‘Plared 15105’ were as affected as those in other cultivars, the newly formed crowns remained healthy, leading to improved plant development and lower overall disease severity. The ability of day-neutral cultivars to form multiple crowns may enhance their ability to tolerate diseases caused by *Neopestalotiopsis* species. Historically, the strawberry industry in Huelva has shown little interest in day-neutral cultivars due to their specific growing requirements (Lauricella, 2021). However, from a breeding perspective, incorporating these cultivars into breeding programs could be beneficial for developing *Neopestalotiopsis*-resistant or tolerant strawberry cultivars.

Environmental conditions also appear to play a role in disease outbreaks. *Neopestalotiopsis* thrives in warm temperatures (Baggio et al., 2021a; Maharachchikumbura et al., 2011), and recent years in Spain have seen higher temperatures during transplanting and establishment, which may have contributed to increased disease severity by exacerbating plant stress. Additionally, commercial strawberry fields in Spain are grown under plastic tunnels, and transplants are initially irrigated using overhead irrigation, which may facilitate disease spread. Since *Neopestalotiopsis* conidia are primarily dispersed by wind and rain splash (Prasannath et al., 2021), overhead irrigation during transplanting may promote spread and favor infection. Adjusting irrigation practices during this critical period could help reduce disease incidence and improve transplant establishment.

Overall, this study advances the taxonomic and pathogenic understanding of *Neopestalotiopsis* species affecting the Spanish strawberry industry, fulfilling our objective to characterize these fungi using molecular techniques and pathogenicity assays. We identified three species, *Neopestalotiopsis* sp., *N. rosae*, and *N. iranensis*, impacting strawberry production fields and nurseries in Spain. Implementing clean transplant programs and selecting more tolerant cultivars may represent effective strategies to mitigate disease caused by these pathogens. Additionally, novel diagnostic tools and continued surveys are needed to monitor species distribution, and breeding programs should account for the current pathogen population structure to develop resistant cultivars.

## Data Availability

All data supporting the findings of this study are available within the manuscript and its supplementary materials. DNA sequences have been deposited in GenBank under the accession numbers listed in **Supplemental Table 1**. Fungal isolates are available from the corresponding authors upon reasonable request.

## References

[B1] Aime M. C. Miller A. N. Aoki T. Bensch K. Cai L. Crous P. . (2021). How to publish a new fungal species, or name, version 3.0. IMA Fungus 12, 11. doi: 10.1186/s43008-021-00063-1 33934723 PMC8091500

[B2] Ávila-Hernández J. G. León-Ramírez C. G. Abraham-Juárez M. D. R. Tlapal-Bolaños B. Olalde-Portugal V. Délano-Frier J. P. . (2025). Neopestalotiopsis spp.: A threat to strawberry production and management. Horticulturae 11, 288. doi: 10.3390/horticulturae1103028830654563

[B3] Avilés M. Castillo S. Bascón J. Zea-Bonilla T. Martín-Sánchez P. M. Pérez-Jiménez R. M. (2008). First report of Macrophomina phaseolina causing crown and root rot of strawberry in Spain. Plant Pathol. J. 57, 382–382. doi: 10.2223/jped.1793 18535739

[B4] Avilés M. Pastrana A. M. Borrero C. (2024). Emerging diseases in Spain strawberry crops: Neopestalotiopsis leaf and crown rot and Fusarium wilt. Plants 13, 3441. doi: 10.3390/plants13233441 PMC11644263 39683234

[B5] Ayoubi N. Soleimani M. (2016). Strawberry fruit rot caused by Neopestalotiopsis iranensis sp. nov. and N. mesopotamica. Curr. Microbiol. 72, 329–336. doi: 10.1007/s00284-015-0955-y 26659835

[B6] Baggio J. S. Forcelini B. B. Whang N.-Y. Ruschel R. G. Mertely J. C. Peres N. A. (2021a). Outbreak of leaf spot and fruit rot in Florida strawberry caused by Neopestalotiopsis spp. Plant Dis. 105, 305–315. doi: 10.1094/pdis-06-20-1290-re 32762327

[B7] Baggio J. S. Marin M. V. Peres N. A. (2021b). Phytophthora crown rot of Florida strawberry: Inoculum sources and thermotherapy of transplants for disease management. Plant Dis. 105, 3496–3502. doi: 10.1094/pdis-11-20-2476-re 34032488

[B8] Benson M. Parker K. (2016). “ PROTOCOL 02-07.1: Rice grain method for Phytophthora inoculum production,” in Laboratory Protocols for Phytophthora Species ( American Phytopathological Society, St. Paul, MN, USA), 1–2. doi: 10.1094/9780890544969.02.07.1

[B9] Borrero C. Castaño R. Avilés M. (2018). First report of Pestalotiopsis clavispora (Neopestalotiopsis clavispora) causing canker and twig dieback on blueberry bushes in Spain. Plant Dis. 101, 1568–1577. doi: 10.1094/pdis-03-19-0473-pdn

[B10] Borrero C. Pastrana A. M. Ordóñez J. Páez J. I. Orta S. Avilés M. (2024). Host range of Phytophthora spp. from berry crops in Huelva, Spain. Plant Dis. 108, 2740–2749. doi: 10.1094/pdis-06-23-1068-re 38616409

[B11] Camili E. C. Carbonari M. Souza N. L. (2002). Caracterização de Pestalotiopsis longisetula e sua patogenicidade em morango. Summa Phytopathol. 28, 213–214.

[B12] Chamorro M. Aguado A. De los Santos B. (2016). First report of root and crown rot caused by Pestalotiopsis clavispora (Neopestalotiopsis clavispora) on strawberry in Spain. Plant Dis. 100, 1495. doi: 10.1094/pdis-11-15-1308-pdn

[B13] Chernomor O. von Haeseler A. Minh B. Q. (2016). Terrace aware data structure for phylogenomic inference from supermatrices. Syst. Biol. 65, 997–1008. doi: 10.1093/sysbio/syw037 27121966 PMC5066062

[B14] Crous P. W. Verkley G. J. M. Groenewald J. Z. Samson R. A. (2009). “ Fungal biodiversity,” in CBS laboratory manual series: 1. Eds. Crous P. W. Verkley G. J. M. Groenewald J. Z. Samson R. A. ( Centraalbureau voor Schimmelcultures, Utrecht, Netherlands), 1–269.

[B15] Dardani G. Martino I. Aloi F. Carli C. Giordano R. Spadaro D. . (2025). Characterization of Neopestalotiopsis species associated with strawberry crown rot in Italy. Agronomy 15, 422. doi: 10.1016/s0022-1139(98)00300-5

[B16] Embaby E. M. (2007). Pestalotia fruit rot on strawberry plants in Egypt. Egypt J. Phytopathol. 35, 99–110. Available online at: https://www.semanticscholar.org/paper/Pestalotia-Fruit-Rot-on-Strawberry-Plants-in-Egypt-Embaby/44b58103c457ea236b4162784ec7609509e69aa1.

[B17] Erdurmuş D. Palacıoğlu G. Erdurmuş G. Bayraktar H. (2022). First report of Neopestalotiopsis rosae causing leaf spot and crown rot of strawberry in Turkey. J. Plant Pathol. 105, 315. doi: 10.1007/s42161-022-01218-8

[B18] Espinoza J. G. Briceño E. X. Keith L. M. Latorre B. A. (2008). Canker and twig dieback of blueberry caused by Pestalotiopsis spp. and a Truncatella sp. in Chile. Plant Dis. 92, 1407–1414. doi: 10.1094/pdis-92-10-1407 30769572

[B19] Essa T. Kamel S. Ismail A. El-Ganainy S. (2018). Characterization and chemical control of Neopestalotiopsis rosae the causal agent of strawberry root and crown rot in Egypt. Egyptian J. Phytopathol. 46, 1–19. doi: 10.21608/ejp.2018.87411

[B20] FAOSTAT (2024). Crops and Livestock Products. Food and Agriculture Organization of the United Nations. Available online at: https://www.fao.org/faostat/en/#data/QCL (Accessed April 14, 2026).

[B21] Gilardi G. Bergeretti F. Gullino M. L. Garibaldi A. (2019). First report of Neopestalotiopsis clavispora causing root and crown rot on strawberry in Italy. Plant Dis. 103, 2959. doi: 10.1094/pdis-91-3-0324c 30780575

[B22] Glass N. L. Donaldson G. C. (1995). Development of primer sets designed for use with the PCR to amplify conserved genes from filamentous ascomycetes. Appl. Environ. Microbiol. 61, 1323–1330. doi: 10.1128/aem.61.4.1323-1330.1995 7747954 PMC167388

[B23] Guba E. F. (1961). Monograph of Pestalotia and Monochaetia. Harvard University Press, Cambridge, MA.

[B24] Guan W. Bonkowski J. Creswell T. Egel D. (2023). Strawberry cultivar susceptibility to Neopestalotiopsis leaf spot in Indiana. Plant Health Prog. 24, 135–139. doi: 10.1094/php-05-22-0049-rs

[B25] Guindon S. Dufayard J.-F. Lefort V. Anisimova M. Hordijk W. Gascuel O. (2010). New algorithms and methods to estimate maximum-likelihood phylogenies: Assessing the performance of PhyML 3.0. Syst. Biol. 59, 307–321. doi: 10.1093/sysbio/syq010 20525638

[B26] Han H. Jang Y. J. Oh Y. Marin M. V. Huguet-Tapia J. Peres N. A. . (2024). Chromosome-scale genome sequence resource for two Neopestalotiopsis spp. isolates with different virulence in strawberry (Fragaria × ananassa). PhytoFrontiers 4, 422–426. doi: 10.1094/phytofr-08-23-0110-a

[B27] Howard C. M. Albregts E. E. (1973). A strawberry fruit rot caused by Pestalotia longisetula. Phytopathology 63, 862–863. doi: 10.1094/phyto-63-862

[B28] Hsu S. Lin Y. Xu Y. Chang H. Chung P. Ariyawansa H. (2022). High-quality genome assembly of Neopestalotiopsis rosae ML1664, the pathogen causing strawberry leaf blight and crown rot. Mol. Plant-Microbe Interact. 35, 949–953. doi: 10.1094/mpmi-04-22-0077-a 36161793

[B29] Index Fungorum. Index Fungorum Partnership (2025). Web. Available online at: https://www.speciesfungorum.org/Names/GSDspecies.asp?RecordID=335984 (Accessed April 14, 2026).

[B30] Kalyaanamoorthy S. Minh B. Q. Wong T. K. F. von Haeseler A. Jermiin L. S. (2017). ModelFinder: Fast model selection for accurate phylogenetic estimates. Nat. Methods 14, 587–589. doi: 10.1038/nmeth.4285 28481363 PMC5453245

[B31] Kenneth R. G. Barkai-Golan R. Netzer D. (1968). A Pestalotia fruit rot of strawberry in Israel. Plant Dis. 52, 472–474.

[B32] Kobayashi T. Ishihara M. Ono Y. (2001). A new species of Pestalosphaeria, the teleomorph of Pestalotiopsis neglecta. Mycoscience 42, 211–216. doi: 10.1127/0029-5035/2006/0083-0151

[B33] Kumar S. Stecher G. Li M. Knyaz C. Tamura K. (2018). MEGAX: Molecular evolutionary genetics analysis across computing platforms. Mol. Biol. Evol. 35, 1547–1549. doi: 10.1093/molbev/msy096 29722887 PMC5967553

[B34] Lauricella V. (2021). Comportamiento de Plantas de Fresa Bajo la Reducción de Aportes Hídricos como Alternativa Sostenible (Huelva: Universidad Internacional de Andalucía; Universidad de Huelva). Máster Oficial Interuniversitario en Tecnología Ambiental.

[B35] Lawrence D. P. Brittain G. D. Aglave B. Sances F. V. (2023). First report of Neopestalotiopsis rosae causing crown and root rot of strawberry in California. Plant Dis. 107, 2. doi: 10.1094/pd-74-0828a

[B36] Liao C. Doilom M. Jeewon R. (2025). Challenges and update on fungal endophytes: Classification, definition, diversity, ecology, evolution and functions. Fungal Diversity 131, 301–367. doi: 10.1007/s13225-025-00550-530311153

[B37] Machín A. González P. Vicente E. Sánchez M. Estelda C. Ghelfi J. . (2019). First report of root and crown rot caused by Neopestalotiopsis clavispora on strawberry in Uruguay. Plant Dis. 103, 2946. doi: 10.1094/PDIS-05-19-0948-PDN

[B38] Mahapatra S. Banerjee J. Kumar K. Pramanik S. Pramanik K. Islam S. . (2018). Leaf spot and fruit rot of strawberry caused by Neopestalotiopsis clavispora in Indo-Gangetic plains of India. Indian Phytopathol. 71, 279–283. doi: 10.1007/s42360-018-0043-x30311153

[B39] Maharachchikumbura S. S. N. Guo L. D. Cai L. Chukeatirote E. Wu W. P. Sun X. . (2012). A multi-locus backbone tree for Pestalotiopsis, with a polyphasic characterization of 14 new species. Fungal Diversity 56, 95–129. doi: 10.1007/s13225-012-0198-130311153

[B40] Maharachchikumbura S. S. N. Guo L. D. Chukeatirote E. Bahkali A. H. Hyde K. D. (2011). Pestalotiopsis—morphology, phylogeny, biochemistry and diversity. Fungal Diversity 50, 167–187. doi: 10.1007/s13225-011-0125-x

[B41] Maharachchikumbura S. S. N. Hyde K. D. Groenewald J. Z. Crous P. W. (2014). Pestalotiopsis revisited. Stud. Mycol. 79, 121–186. doi: 10.1016/j.simyco.2014.09.005 PMC4255583 25492988

[B42] Marin M. V. Wang N.-Y. Turechek W. W. Peres N. A. (2025). Thermotherapy via aerated steam is an effective alternative for nonchemical management of cryptic infection of strawberry by Colletotrichum acutatum. Plant Dis. 109, 1745–1752. doi: 10.1094/pdis-03-24-0700-re 39970110

[B43] McNally J. Prapagar K. Goldenhar K. Pate E. Shan S. Kalischuk M. (2023). First report of an aggressive species of Neopestalotiopsis affecting strawberry in Canada. New Dis. Rep. 48, e12210. doi: 10.1002/ndr2.1221041531421

[B44] McQuilken J. Prapagar K. Goldenhar K. Pate E. Shan S. Kalischuk M. (2004). Biology and integrated control of Pestalotiopsis on container-grown ericaceous crops. Pest Manage. Sci. 60, 135–142. doi: 10.1002/ps.792 14971679

[B45] Medina J. J. (2008). La fresa en Huelva 15–47. ISBN: 84-8474-222-9.

[B46] Minh B. Q. Schmidt H. A. Chernomor O. Schrempf D. Woodhams M. D. von Haeseler A. . (2020). IQ-TREE 2: New models and efficient methods for phylogenetic inference in the genomic era. Mol. Biol. Evol. 37, 1530–1534. doi: 10.1093/molbev/msaa015 32011700 PMC7182206

[B47] Morales-Mora L. A. Martínez-Salgado S. J. Valencia de Ita M. A. Andrade-Hoyos P. Silva-Rojas H. V. Romero-Arenas O. (2019). First report of leaf spot and anthracnosis caused by Pestalotiopsis sp. on strawberry in Puebla, Mexico. Plant Dis. 103, 2668. doi: 10.1094/pdis-05-19-1010-pdn

[B48] Mouden N. Benkirane R. Ouazzani Touhami A. Douira A. (2014). Pathogenic capacity of Pestalotia longisetula Guba reported for the first time on strawberry (Fragaria ananassa Duch.) in Morocco. Int. J. Pure Appl. Bioscience 2 (4), 132–141. Available online at: https://www.ijpab.com/form/2014%20Volume%202,%20issue%204/IJPAB-2014-2-4-132-141.pdf.

[B49] Naser-Khdour S. Minh B. Q. Zhang W. Stone E. A. Lanfear R. (2019). The prevalence and impact of model violations in phylogenetic analysis. Genome Biol. Evol. 11, 3341–3352. doi: 10.1093/gbe/evz193 31536115 PMC6893154

[B50] O'Donnell K. Cigelnik E. (1997). Two divergent intragenomic rDNA ITS2 types within a monophyletic lineage of the fungus Fusarium are nonorthologous. Mol. Phylogenet. Evol. 103, 16. doi: 10.1006/mpev.1996.0376 9007025

[B51] Obregon V. G. Meneguzzi N. G. Ibañez J. M. Lattar T. E. Kirschbaum D. S. (2018). First report of Neopestalotiopsis clavispora causing root and crown rot on strawberry plants in Argentina. Plant Dis. 102, 1856. doi: 10.2139/ssrn.5260939

[B52] Pastrana A. M. Basallote-Ureba M. J. Aguado A. Capote N. (2017). Potential inoculum sources and incidence of strawberry soilborne pathogens in Spain. Plant Dis. 101, 751–760. doi: 10.1094/pdis-08-16-1177-re 30678576

[B53] Prasannath K. Galea V. Akinsanmi O. (2021). Molecular methods for the detection and quantification of Pestalotiopsis and Neopestalotiopsis inoculum associated with macadamia. Plant Pathol. 70, 1209–1218. doi: 10.1111/ppa.1337140046247

[B54] Prieto-Rodríguez P. Pastrana A. M. Borrero C. Avilés M. (2024). First report of Fusarium oxysporum f. sp. fragariae race 1 causing fusarium wilt of strawberry (Fragaria × ananassa) in the Alentejo region of Portugal. Plant Dis. 108, 10. doi: 10.1097/00007890-200004150-0006133079771

[B55] Rebello C. Wang N. Marin M. Baggio J. Peres N. (2023). Detection and species differentiation of Neopestalotiopsis spp. from strawberry (Fragaria x ananassa) in Florida using a high-resolution melting (HRM) analysis. PhytoFrontiers 3, 156–163. doi: 10.1094/phytofr-03-22-0034-fi

[B56] Rebollar-Alviter A. Silva-Rojas H. V. Fuentes-Aragón D. Acosta-González U. Martínez-Ruiz M. Parra-Robles B. E. (2020). An emerging strawberry fungal disease associated with root rot, crown rot and leaf spot caused by Neopestalotiopsis rosae in Mexico. Plant Dis. 104, 2054–2059. doi: 10.1094/pdis-11-19-2493-sc 32515689

[B57] Rehner S. A. Buckley E. (2005). A Beauveria phylogeny inferred from nuclear ITS and EF1-α sequences: evidence for cryptic diversification and links to Cordyceps teleomorphs. Mycologia 97 (1), 84–98. doi: 10.1080/15572536.2006.11832842 16389960

[B58] Ronquist F. Teslenko M. van der Mark P. Ayres D. L. Darling A. Höhna S. . (2012). MrBayes 3.2: Efficient Bayesian phylogenetic inference and model choice across a large model space. Syst. Biol. 61, 539–542. doi: 10.1093/sysbio/sys029 22357727 PMC3329765

[B59] Rotondo F. Klass T. L. Scott K. McCartney M. Jacobs J. M. Lewis Ivey M. L. (2023). First report of Neopestalotiopsis disease in Ohio caused by an emerging and novel species of Neopestalotiopsis on strawberry. Plant Dis. 107, 3. doi: 10.1094/pdis-04-24-0778-pdn

[B60] Shoemaker R. A. Simpson J. A. (1981). A new species of Pestalosphaeria on pine with comments on the generic placement of the anamorph. Can. J. Bot. 59, 986–991. doi: 10.5479/si.00963801.102-3299.231

[B61] Sinclair J. B. Dhingra O. D. (1995). Basic Plant Pathology Methods (Boca Raton: CRC Press). doi: 10.1201/9781315138138

[B62] Sultana S. Sikder M. M. Ahmmed S. M. Alam N. (2022). Neopestalotiopsis chrysea causing leaf spot disease of strawberry plants in Bangladesh. J. Plant Sci. 17, 66–74. doi: 10.3923/jps.2022.66.74

[B63] Sun Q. Harishchandra D. Jia J. Zuo Q. Zhang G. Wang Q. . (2021). Role of Neopestalotiopsis rosae in causing root rot of strawberry in Beijing, China. Crop Prot. 147, 105710. doi: 10.14257/ijmue.2014.9.11.06

[B64] Tamura K. Stecher G. Kumar S. (2021). MEGA11: Molecular evolutionary genetics analysis version 11. Mol. Biol. Evol. 38, 3022–3027. doi: 10.1093/molbev/msab120 33892491 PMC8233496

[B65] White T. J. Bruns T. Lee S. Taylor J. (1990). “ Amplification and direct sequencing of fungal ribosomal RNA genes for phylogenetics,” in PCR protocols: a guide to methods and applications ( Academic Press, New York), 315–322.

[B66] Wu H.-Y. Tsai C.-Y. Wu Y.-M. Ariyawansa H.-A. Chung C.-L. Chung P.-C. (2020). First report of Neopestalotiopsis rosae causing leaf blight and crown rot on strawberry in Taiwan. Plant Dis. 105, 487. doi: 10.1016/j.cllc.2025.03.007 40268596

[B67] Zhao J. N. (2016). Pestalotiopsis clavispora causing leaf spot on strawberry. Mycosystema 35, 114–120.

[B68] Zuniga A. I. Baggio J. S. Peres N. A. (2024). A semi-selective medium to evaluate over-summering survival of Neopestalotiopsis sp. in Florida strawberry fields. Plant Dis. 108, 2096–2103. doi: 10.1094/pdis-11-23-2304-re 38411605

