# Correction: Molecular characterization reveals three *Neopestalotiopsis* species causing strawberry disease outbreaks in Spain

**DOI:** 10.3389/fpls.2026.1997554

**Published:** 2026-10-08

**Authors:** Patricia López, Ana M. Pastrana, Marcus V. Marin, Celia Borrero, Natalia A. Peres, Manuel Avilés

**Affiliations:** 1 Departamento de Agronomía, ETSIA-Universidad de Sevilla, Sevilla, Spain; 2 University of California Agriculture and Natural Resources, Cooperative Extension, Holtville, CA, United States; 3 Plant Pathology Department, Gulf Coast Research and Education Center, University of Florida, Wimauma, FL, United States

**Keywords:** emerging pathogens, *Neopestalotiopsis iranensis*, *Neopestalotiopsis rosae*, phylogenetic analysis, strawberry diseases

An error was made in the citation of **Supplementary Material**, and in the **Supplementary Files** themselves as originally uploaded. The file originally posted as “**Supplementary Table 1**” did not correspond to what is cited under that name in the text – it contained the two tables that should have been **Supplementary Tables 2**, **3** (ANOVA results for incidence and severity, respectively). The correct **Supplementary Table 1**, containing GenBank accession numbers for the sequenced isolates, was never uploaded.

All supplementary material files have been replaced accordingly: **Supplementary Table 1** (GenBank accession numbers), **Supplementary Table 2** (factorial ANOVA, incidence), and **Supplementary Table 3** (factorial ANOVA, severity, and test of separation of means).

A correction has been made to the section **Results**, subsection *Pathogenicity test*, paragraph 1. This originally read: “Non-inoculated control plants remained healthy in all assays and were not included in the statistical analysis. In the factorial ANOVA for the incidence of crown and leaf inoculations, the factors included were: isolate, cultivar, assay, and the interaction between isolate × cultivar, with only isolate and cultivar being significant (**Supplementary Table 1**). In the factorial ANOVA for severity of crown and leaf inoculations, the factors were: isolate, cultivar, assay, block nested in the assay, and the interaction between isolate × cultivar, and only isolate and cultivar were significant (**Supplementary Table 2**). Therefore, we present the effects of these two main factors on both dependent variables, after the corresponding test of separation of means (**Table 2**).” This has been updated to read:

"Non-inoculated control plants remained healthy in all assays and were not included in the statistical analysis. In the factorial ANOVA for the incidence of crown and leaf inoculations, the factors included were: isolate, cultivar, assay, and the interaction between isolate × cultivar, with only isolate and cultivar being significant (**Supplementary Table 2**). In the factorial ANOVA for severity of crown and leaf inoculations, the factors were: isolate, cultivar, assay, block nested in the assay, and the interaction between isolate × cultivar, and only isolate and cultivar were significant (**Supplementary Table 3**). Therefore, we present the effects of these two main factors on both dependent variables, after the corresponding test of separation of means (**Table 2**)."

The original version of this article has been updated.

