# Supplementary material for: Molecular characterization reveals three Neopestalotiopsis species causing strawberry disease outbreaks in Spain
Source: Front Plant Sci. 2026 May 11;17:1830265. doi: 10.3389/fpls.2026.1830265 (PMC13199264; doi:10.3389/fpls.2026.1830265)
Supplement: Supplementary file 2 [file DataSheet1.pdf]

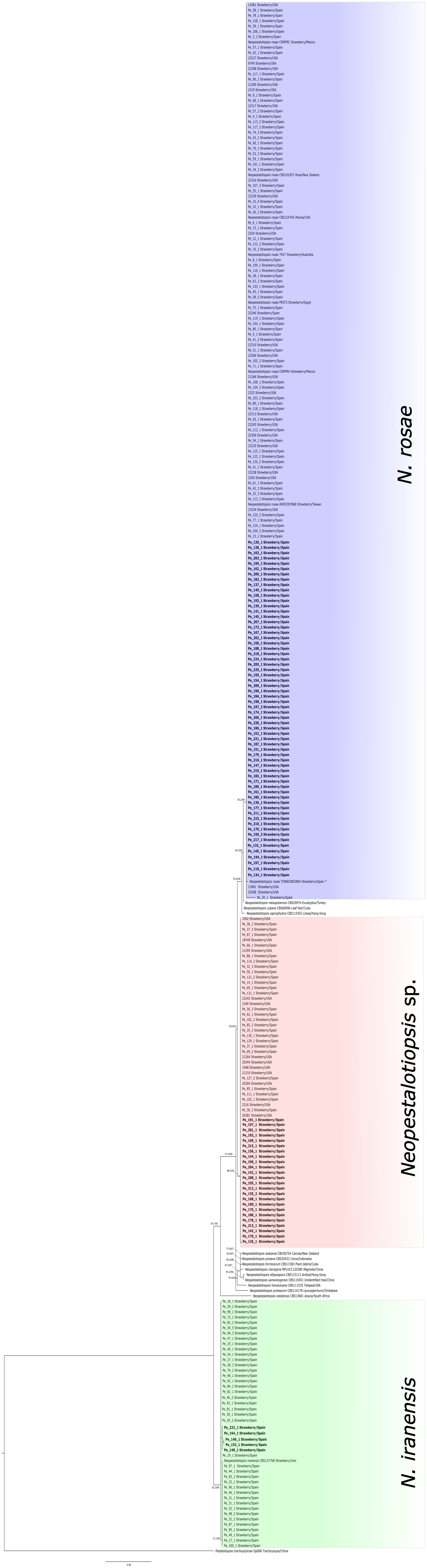
N. rosae
Neopestalotiopsis sp.
N. iranensis
