# Supplementary material for: Molecular characterization reveals three Neopestalotiopsis species causing strawberry disease outbreaks in Spain
Source: Front Plant Sci. 2026 May 11;17:1830265. doi: 10.3389/fpls.2026.1830265 (PMC13199264; doi:10.3389/fpls.2026.1830265)
Supplement: Supplementary file 3 [file DataSheet2.pdf]

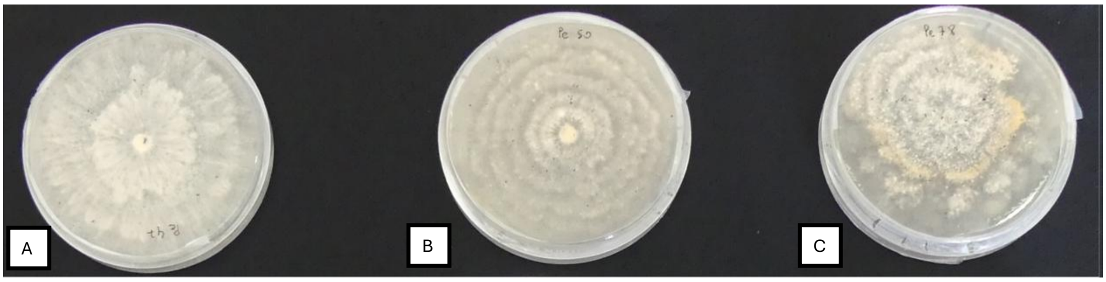

Figure S2. Reverse view of culture plates of three *Neopestalotiopsis* species. **A.** Colonies of *N. iranensis* were white to pale yellow. **B.** Colonies of *Neopestalotiopsis* sp. are similar to those of *N. iranensis*, ranging from white to pale yellow. **C.** Colonies of *N. rosae* were pale luteous to orange.
