# Supplementary material for: Molecular characterization reveals three Neopestalotiopsis species causing strawberry disease outbreaks in Spain
Source: Front Plant Sci. 2026 May 11;17:1830265. doi: 10.3389/fpls.2026.1830265 (PMC13199264; doi:10.3389/fpls.2026.1830265)
Supplement: Supplementary file 4 [file DataSheet3.pdf]

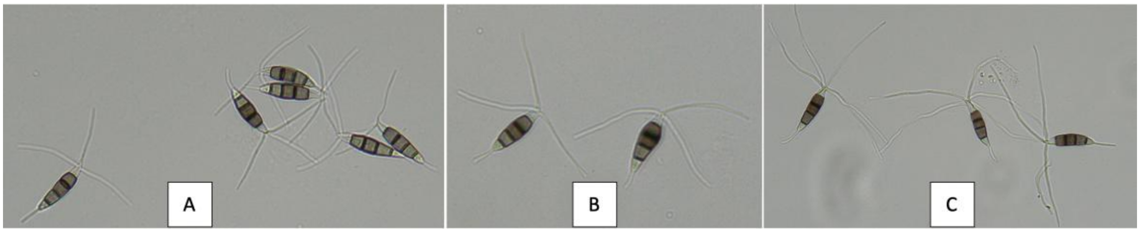

Figure S3. Number of apical appendages in conidia of different *Neopestalotiopsis* species. **A.** *Neopestalotiopsis rosae* conidia had two to four (predominantly three) apical appendages. **B.** *Neopestalotiopsis* sp. conidia had two to four (predominantly three) apical appendages. **C.** *Neopestalotiopsis iranensis* conidia had three to five (predominantly four) apical appendages.
