# Supplementary material for: Molecular characterization reveals three Neopestalotiopsis species causing strawberry disease outbreaks in Spain
Source: Front Plant Sci. 2026 May 11;17:1830265. doi: 10.3389/fpls.2026.1830265 (PMC13199264; doi:10.3389/fpls.2026.1830265)
Supplement: Supplementary file 6 [file Table1.docx]

**Supplemental Table 1.** *Neopestalotiopsis* isolates from Spain characterized in this study.

| **Specie** ^**q**^ | **Isolates** ^**r**^ | **Month-Year** ^**s**^ | **Origin** ^**t**^ | **Host** ^**u**^ | **Source** ^**v**^ | **Cultivar** ^**w**^ | **ITS** ^**x**^ | **TEF** ^**y**^ | **TUB** ^**z**^ |
| --- | --- | --- | --- | --- | --- | --- | --- | --- | --- |
| *N. rosae* | **Pe_3_2** | Sep-2010 | N/A | *Vaccinium corymbosun* | N/A | N/A | PQ771775 | PV010375 | PV010489 |
| *N. rosae* | **Pe_4_1** | Sep-2010 | N/A | *Vaccinium corymbosun* | N/A | N/A | PQ771776 | PV010376 | PV010490 |
| *N. rosae* | **Pe_5_1** | Sep-2010 | N/A | *Vaccinium corymbosun* | N/A | N/A | PQ771777 | PV010377 | PV010491 |
| *N. rosae* | **Pe_6_1** | Sep-2013 | BPF | *Vaccinium corymbosun* | N/A | N/A | PQ771713 | PV010378 | PV010492 |
| *N. rosae* | **Pe_8_1** | Oct-2013 | BPF | *Vaccinium corymbosun* | N/A | N/A | PQ771778 | PV010379 | PV010493 |
| *N. rosae* | **Pe_9_1** | Oct-2013 | BPF | *Vaccinium corymbosun* | N/A | N/A | PQ771779 | PV010380 | PV010494 |
| *N. rosae* | **Pe_10_1** | Feb-2014 | N/A | *Vaccinium corymbosun* | N/A | N/A | PQ771780 | PV010381 | PV010495 |
| *N. rosae* | **Pe_10_4** | Feb-2014 | N/A | *Vaccinium corymbosun* | N/A | N/A | PQ771781 | PV010382 | PV010496 |
| *N. rosae* | **Pe_12_1** | Dec-2020 | SPF | *Fragaria x ananassa* | N/A | N/A | PQ771782 | PV010383 | PV010497 |
| *N. rosae* | **Pe_15_1** | Nov-2021 | SPF | *Fragaria x ananassa* | N/A | N/A | PQ771714 | PV010384 | PV010498 |
| *N. rosae* | **Pe_20_1** | Nov-2021 | SPF | *Fragaria x ananassa* | N/A | N/A | PQ771783 | PV010385 | PV032027 |
| *N. rosae* | **Pe_26_2** | Nov-2021 | NRP (CL) | *Fragaria x ananassa* | N/A | 277 FNM | PQ771784 | PV010386 | PV010499 |
| *N. rosae* | **Pe_33_3** | Jan-2022 | SPF | *Fragaria x ananassa* | N/A | N/A | PQ771785 | PV010387 | PV010500 |
| *N. rosae* | **Pe_34_2** | Jan-2022 | SPF | *Fragaria x ananassa* | N/A | N/A | PQ771715 | PV010388 | PV010501 |
| *N. rosae* | **Pe_38_1** | Jan-2022 | SPF | *Fragaria x ananassa* | N/A | N/A | PQ771716 | PV010389 | PV010502 |
| *N. rosae* | **Pe_39_1** | Jan-2022 | SPF | *Fragaria x ananassa* | N/A | N/A | PQ771717 | PV010390 | PV010503 |
| *N. rosae* | **Pe_41_1** | Jan-2022 | SPF | *Fragaria x ananassa* | N/A | N/A | PQ771786 | PV010391 | PV010504 |
| *N. rosae* | **Pe_41_2** | Jan-2022 | SPF | *Fragaria x ananassa* | N/A | N/A | PQ771787 | PV010392 | PV010505 |
| *N. rosae* | **Pe_42_1** | Jan-2022 | SPF | *Fragaria x ananassa* | N/A | N/A | PQ771788 | PV010393 | PV010506 |
| *N. rosae* | **Pe_42_3** | Jan-2022 | SPF | *Fragaria x ananassa* | N/A | N/A | PQ771789 | PV010394 | PV010507 |
| *N. rosae* | **Pe_43_2** | May-2022 | SPF | *Fragaria x ananassa* | N/A | N/A | PQ771790 | PV010395 | PV010508 |
| *N. rosae* | **Pe_45_1** | Feb-2022 | SPF | *Fragaria x ananassa* | Crown | Primoris | PQ771718 | PV010396 | PV010509 |
| *N. rosae* | **Pe_51_1** | Feb-2022 | SPF | *Fragaria x ananassa* | Crown | Leticia | PQ771791 | PV010397 | PV010510 |
| *N. rosae* | **Pe_53_3** | Apr-2022 | SPF | *Fragaria x ananassa* | Root | Marimbella | PQ771719 | PV010398 | PV010511 |
| *N. rosae* | **Pe_54_1** | Apr-2022 | SPF | *Fragaria x ananassa* | Root | Marimbella | PQ771792 | PV010399 | PV010512 |
| *N. rosae* | **Pe_55_1** | Apr-2022 | SPF | *Fragaria x ananassa* | Crown | Leticia | PQ771720 | PV010400 | PV010513 |
| *N. rosae* | **Pe_57_1** | Apr-2022 | SPF | *Fragaria x ananassa* | Crown | Leticia | PQ771793 | PV010401 | PV010514 |
| *N. rosae* | **Pe_57_2** | Apr-2022 | SPF | *Fragaria x ananassa* | Crown | Leticia | PQ771794 | PV010402 | PV010515 |
| *N. rosae* | **Pe_58_1** | Apr-2022 | SPF | *Fragaria x ananassa* | Crown | Leticia | PQ771795 | PV010403 | PV010516 |
| *N. rosae* | **Pe_58_5** | Apr-2022 | SPF | *Fragaria x ananassa* | Crown | Leticia | PQ771796 | PV010404 | PV010517 |
| *N. rosae* | **Pe_59_1** | Apr-2022 | SPF | *Fragaria x ananassa* | Crown | Leticia | PQ771797 | PV010405 | PV010518 |
| *N. rosae* | **Pe_60_1** | Apr-2022 | SPF | *Fragaria x ananassa* | Crown | Leticia | PQ771798 | PV010406 | PV010519 |
| *N. rosae* | **Pe_61_1** | Apr-2022 | SPF | *Fragaria x ananassa* | Root | Amparo | PQ771799 | PV010407 | PV010520 |
| *N. rosae* | **Pe_63_2** | Apr-2022 | SPF | *Fragaria x ananassa* | Root | Leticia | PQ771721 | PV010408 | PV010521 |
| *N. rosae* | **Pe_65_1** | Apr-2022 | SPF | *Fragaria x ananassa* | Crown | Leticia | PQ771800 | PV010409 | PV010523 |
| *N. rosae* | **Pe_68_1** | Mar-2022 | SPF | *Fragaria x ananassa* | Crown | PALO3B_2 | PQ771801 | PV010410 | PV010524 |
| *N. rosae* | **Pe_70_3** | Mar-2022 | SPF | *Fragaria x ananassa* | Crown | LEO1B_5 | PQ771802 | PV010411 | PV010525 |
| *N. rosae* | **Pe_71_1** | Mar-2022 | SPF | *Fragaria x ananassa* | Crown | LEO3B_1 | PQ771803 | PV010412 | PV010526 |
| *N. rosae* | **Pe_73_1** | Apr-2022 | SPF | *Fragaria x ananassa* | Crown | Palmeritas | PQ771804 | PV010413 | PV010527 |
| *N. rosae* | **Pe_74_3** | Apr-2022 | SPF | *Fragaria x ananassa* | Crown | Palmeritas | PQ771722 | PV010414 | PV010528 |
| *N. rosae* | **Pe_75_1** | Apr-2022 | SPF | *Fragaria x ananassa* | Root | Palmeritas | PQ771805 | PV010415 | PV010529 |
| *N. rosae* | **Pe_76_2** | Apr-2022 | SPF | *Fragaria x ananassa* | Root | Palmeritas | PQ771806 | PV010416 | PV010530 |
| *N. rosae* | **Pe_77_1** | Apr-2022 | SPF | *Fragaria x ananassa* | Crown | Palmeritas | PQ771723 | PV010417 | PV010531 |
| *N. rosae* | **Pe_78_1** | Apr-2022 | SPF | *Fragaria x ananassa* | Crown | Palmeritas | PQ771724 | PV010418 | PV010532 |
| *N. rosae* | **Pe_80_1** | Apr-2022 | SPF | *Fragaria x ananassa* | Crown | Calinda | PQ771807 | PV010419 | PV010533 |
| *N. rosae* | **Pe_89_1** | Apr-2022 | SPF | *Fragaria x ananassa* | Crown | Palmeritas | PQ771725 | PV010420 | PV010535 |
| *N. rosae* | **Pe_90_2** | Apr-2022 | SPF | *Fragaria x ananassa* | Crown | Calinda | PQ771726 | PV010421 | PV010536 |
| *N. rosae* | **Pe_101_1** | May-2022 | N/A | *Hordeum vulgare L.* | N/A | N/A | PQ771808 | PV010422 | PV010537 |
| *N. rosae* | **Pe_103_1** | Jun-2022 | SPF | *Fragaria x ananassa* | Root | Leticia | PQ771809 | PV010423 | PV010538 |
| *N. rosae* | **Pe_103_2** | Jun-2022 | SPF | *Fragaria x ananassa* | Root | Leticia | PQ771810 | PV010424 | PV010539 |
| *N. rosae* | **Pe_104_1** | Jun-2022 | SPF | *Fragaria x ananassa* | Root | Palmeritas | PQ771811 | PV010425 | PV010540 |
| *N. rosae* | **Pe_104_2** | Jun-2022 | SPF | *Fragaria x ananassa* | Root | Palmeritas | PQ771812 | PV010426 | PV010541 |
| *N. rosae* | **Pe_104_3** | Jun-2022 | SPF | *Fragaria x ananassa* | Root | Palmeritas | PQ771813 | PV010427 | PV010542 |
| *N. rosae* | **Pe_105_2** | Jun-2022 | SPF | *Fragaria x ananassa* | Crown | Palmeritas | PQ771814 | PV010428 | PV010543 |
| *N. rosae* | **Pe_106_1** | Jun-2022 | SPF | *Fragaria x ananassa* | Crown | Palmeritas | PQ771727 | PV010429 | PV010544 |
| *N. rosae* | **Pe_107_3** | Jun-2022 | SPF | *Fragaria x ananassa* | Root | Palmeritas | PQ771815 | PV010430 | PV010545 |
| *N. rosae* | **Pe_108_1** | Jun-2022 | SPF | *Fragaria x ananassa* | Root | Palmeritas | PQ771816 | PV010431 | PV010546 |
| *N. rosae* | **Pe_109_1** | Jun-2022 | SPF | *Fragaria x ananassa* | Root | Palmeritas | PQ771817 | PV010432 | PV010547 |
| ***N. rosae*** | **Pe_110_1** | **Sep-2022** | **CD** | ***Fragaria x ananassa*** | **Crown** | **Leticia** | **N/A** | **N/A** | **PV010564** |
| *N. rosae* | **Pe_112_1** | Jun-2022 | SPF | *Fragaria x ananassa* | Root | Palmeritas | PQ771818 | PV010433 | PV010548 |
| *N. rosae* | **Pe_113_2** | Jun-2022 | SPF | *Fragaria x ananassa* | Root | Palmeritas | PQ771819 | PV010434 | PV010549 |
| *N. rosae* | **Pe_113_3** | Jun-2022 | SPF | *Fragaria x ananassa* | Root | Palmeritas | PQ771820 | PV010435 | PV010550 |
| *N. rosae* | **Pe_115_1** | Jun-2022 | SPF | *Fragaria x ananassa* | Crown | Palmeritas | PQ771728 | PV010436 | PV010551 |
| *N. rosae* | **Pe_116_1** | Jun-2022 | BPF | *Vaccinium corymbosun* | N/A | Andrea | PQ771821 | PV010437 | PV010552 |
| *N. rosae* | **Pe_117_1** | Jun-2022 | BPF | *Vaccinium corymbosun* | N/A | Elaine | PQ771822 | PV010438 | PV010553 |
| *N. rosae* | **Pe_117_2** | Jun-2022 | BPF | *Vaccinium corymbosun* | N/A | Elaine | PQ771823 | PV010439 | PV010554 |
| *N. rosae* | **Pe_118_1** | Jul-2022 | NRP (G) | *Fragaria x ananassa* | Crown | Leyre | PQ771824 | PV010440 | PV010555 |
| *N. rosae* | **Pe_119_1** | Jul-2022 | NRP (G) | *Fragaria x ananassa* | Crown | Leyre | PQ771729 | PV010441 | PV010556 |
| ***N. rosae*** | **Pe_120_1** | **Sep-2022** | **NRP (CL)** | ***Fragaria x ananassa*** | **Petiole** | N/A | **N/A** | **N/A** | **PV010565** |
| *N. rosae* | **Pe_122_2** | Jul-2022 | NRP (G) | *Fragaria x ananassa* | Crown | Leyre | PQ771730 | PV010442 | PV010557 |
| *N. rosae* | **Pe_123_1** | Jul-2022 | NRP (G) | *Fragaria x ananassa* | Crown | Leyre | PQ771731 | PV010443 | PV010558 |
| *N. rosae* | **Pe_124_1** | Jul-2022 | NRP (G) | *Fragaria x ananassa* | Crown | Leyre | PQ771732 | PV010444 | PV010559 |
| *N. rosae* | **Pe_125_2** | Jul-2022 | NRP (G) | *Fragaria x ananassa* | Crown | Leyre | PQ771733 | PV010445 | PV010560 |
| *N. rosae* | **Pe_126_2** | Jul-2022 | NRP (G) | *Fragaria x ananassa* | Crown | Leyre | PQ771825 | PV010446 | PV010561 |
| *N. rosae* | **Pe_128_1** | Jul-2022 | NRP (G) | *Fragaria x ananassa* | Crown | Leyre | PQ771734 | PV010447 | PV010562 |
| ***N. rosae*** | **Pe_132_1** | **Sep-2022** | **NRP (CL)** | ***Fragaria x ananassa*** | **Petiole** | **N/A** | **N/A** | **N/A** | **PV010566** |
| ***N. rosae*** | **Pe_134_1** | **Sep-2022** | **NRP (CL)** | ***Fragaria x ananassa*** | **Petiole** | **N/A** | **N/A** | **N/A** | **PV010567** |
| ***N. rosae*** | **Pe_136_1** | **Sep-2022** | **NRP (CL)** | ***Fragaria x ananassa*** | **Petiole** | **N/A** | **N/A** | **N/A** | **PV010568** |
| ***N. rosae*** | **Pe_137_1** | **Sep-2022** | **NRP (CL)** | ***Fragaria x ananassa*** | **Petiole** | **N/A** | **N/A** | **N/A** | **PV010569** |
| ***N. rosae*** | **Pe_138_1** | **Oct-2022** | **SPF** | ***Fragaria x ananassa*** | **Root** | **Plared 15105** | **N/A** | **N/A** | **PV010570** |
| ***N. rosae*** | **Pe_139_1** | **Oct-2022** | **SPF** | ***Fragaria x ananassa*** | **Crown** | **Plared 15105** | **N/A** | **N/A** | **PV010571** |
| ***N. rosae*** | **Pe_140_1** | **Oct-2022** | **SPF** | ***Fragaria x ananassa*** | **Crown** | **Plared 15105** | **N/A** | **N/A** | **PV010572** |
| ***N. rosae*** | **Pe_141_1** | **Oct-2022** | **NRP (CL)** | ***Fragaria x ananassa*** | **Leaf** | **N/A** | **N/A** | **N/A** | **PV010573** |
| ***N. rosae*** | **Pe_145_1** | **Oct-2022** | **NRP (CL)** | ***Fragaria x ananassa*** | **Leaf** | **N/A** | **N/A** | **N/A** | **PV010574** |
| ***N. rosae*** | **Pe_147_1** | **Oct-2022** | **NRP (CL)** | ***Fragaria x ananassa*** | **Crown** | **Beauty** | **N/A** | **N/A** | **PV010575** |
| ***N. rosae*** | **Pe_149_1** | **Oct-2022** | **NRP (CL)** | ***Fragaria x ananassa*** | **Crown** | **Victory** | **N/A** | **N/A** | **PV010576** |
| ***N. rosae*** | **Pe_150_1** | **Oct-2022** | **NRP (G)** | ***Fragaria x ananassa*** | **Crown** | **Palmeritas** | **N/A** | **N/A** | **PV010577** |
| ***N. rosae*** | **Pe_150_2** | **Oct-2022** | **NRP (CL)** | ***Fragaria x ananassa*** | **Crown** | **Palmeritas** | **N/A** | **N/A** | **PV010578** |
| ***N. rosae*** | **Pe_151_1** | **Oct-2022** | **NRP (CL)** | ***Fragaria x ananassa*** | **Crown** | **Fortuna** | **N/A** | **N/A** | **PV010579** |
| ***N. rosae*** | **Pe_152_1** | **Oct-2022** | **NRP (CL)** | ***Fragaria x ananassa*** | **Crown** | **Fortuna** | **N/A** | **N/A** | **PV010580** |
| ***N. rosae*** | **Pe_154_1** | **Dec-2022** | **NRP (CL)** | ***Fragaria x ananassa*** | **Crown** | **Fortuna** | **N/A** | **N/A** | **PV010581** |
| ***N. rosae*** | **Pe_158_1** | **Dec-2022** | **NRP (CL)** | ***Fragaria x ananassa*** | **Root** | **Leticia** | **N/A** | **N/A** | **PV010582** |
| ***N. rosae*** | **Pe_159_1** | **Dec-2022** | **SPF** | ***Fragaria x ananassa*** | **Crown** | **vk-19-138** | **N/A** | **N/A** | **PV010583** |
| ***N. rosae*** | **Pe_161_1** | **Dec-2022** | **CD** | ***Fragaria x ananassa*** | **Crown** | **Leticia** | **N/A** | **N/A** | **PV010584** |
| ***N. rosae*** | **Pe_162_1** | **Dec-2022** | **NRP (CL)** | ***Fragaria x ananassa*** | **Crown** | **Fortuna** | **N/A** | **N/A** | **PV010585** |
| ***N. rosae*** | **Pe_163_1** | **Dec-2022** | **NRP (CL)** | ***Fragaria x ananassa*** | **Crown** | **Fortuna** | **N/A** | **N/A** | **PV010586** |
| ***N. rosae*** | **Pe_167_1** | **Dec-2022** | **NRP (CL)** | ***Fragaria x ananassa*** | **Crown** | **Palmeritas** | **N/A** | **N/A** | **PV010587** |
| ***N. rosae*** | **Pe_171_1** | **Dec-2022** | **NRP (G)** | ***Fragaria x ananassa*** | **Crown** | **Leticia** | **N/A** | **N/A** | **PV010588** |
| ***N. rosae*** | **Pe_173_1** | **Dec-2022** | **NRP (G)** | ***Fragaria x ananassa*** | **Crown** | **Leticia** | **N/A** | **N/A** | **PV010589** |
| ***N. rosae*** | **Pe_174_1** | **Oct-2022** | **SPF** | ***Fragaria x ananassa*** | **Crown** | **vk-19-138** | **N/A** | **N/A** | **PV010590** |
| ***N. rosae*** | **Pe_176_1** | **Dec-2022** | **NRP (G)** | ***Fragaria x ananassa*** | **Crown** | **Cleopatra** | **N/A** | **N/A** | **PV010591** |
| ***N. rosae*** | **Pe_177_1** | **Nov-2022** | **SPF** | ***Fragaria x ananassa*** | **Root** | **N/A** | **N/A** | **N/A** | **PV010592** |
| ***N. rosae*** | **Pe_179_1** | **Feb-2023** | **SPF** | ***Fragaria x ananassa*** | **Crown** | **Leticia** | **N/A** | **N/A** | **PV010593** |
| ***N. rosae*** | **Pe_182_1** | **Feb-2023** | **SPF** | ***Fragaria x ananassa*** | **Leaf** | **Leticia** | **N/A** | **N/A** | **PV010594** |
| ***N. rosae*** | **Pe_183_1** | **Feb-2023** | **SPF** | ***Fragaria x ananassa*** | **Crown** | **Leticia** | **N/A** | **N/A** | **PV010595** |
| ***N. rosae*** | **Pe_184_1** | **Feb-2023** | **SPF** | ***Fragaria x ananassa*** | **Leaf** | **Leticia** | **N/A** | **N/A** | **PV010596** |
| ***N. rosae*** | **Pe_185_1** | **Feb-2023** | **SPF** | ***Fragaria x ananassa*** | **Root** | **Leticia** | **N/A** | **N/A** | **PV010597** |
| ***N. rosae*** | **Pe_186_1** | **Feb-2023** | **SPF** | ***Fragaria x ananassa*** | **Crown** | **Leticia** | **N/A** | **N/A** | **PV010598** |
| ***N. rosae*** | **Pe_187_1** | **Feb-2023** | **SPF** | ***Fragaria x ananassa*** | **Crown** | **Leticia** | **N/A** | **N/A** | **PV010599** |
| ***N. rosae*** | **Pe_188_1** | **Feb-2023** | **SPF** | ***Fragaria x ananassa*** | **Root** | **Leticia** | **N/A** | **N/A** | **PV010600** |
| ***N. rosae*** | **Pe_189_1** | **Feb-2023** | **SPF** | ***Fragaria x ananassa*** | **Crown** | **Leticia** | **N/A** | **N/A** | **PV010601** |
| ***N. rosae*** | **Pe_190_1** | **Feb-2023** | **SPF** | ***Fragaria x ananassa*** | **Crown** | **Leticia** | **N/A** | **N/A** | **PV010602** |
| ***N. rosae*** | **Pe_193_1** | **Feb-2023** | **SPF** | ***Fragaria x ananassa*** | **Petiole** | **Leticia** | **N/A** | **N/A** | **PV010603** |
| ***N. rosae*** | **Pe_194_1** | **Feb-2023** | **SPF** | ***Fragaria x ananassa*** | **Crown** | **Leticia** | **N/A** | **N/A** | **PV010604** |
| ***N. rosae*** | **Pe_197_1** | **Feb-2023** | **SPF** | ***Fragaria x ananassa*** | **Crown** | **Leticia** | **N/A** | **N/A** | **PV010605** |
| ***N. rosae*** | **Pe_197_2** | **Feb-2023** | **SPF** | ***Fragaria x ananassa*** | **Crown** | **Leticia** | **N/A** | **N/A** | **PV010606** |
| ***N. rosae*** | **Pe_198_1** | **Feb-2023** | **SPF** | ***Fragaria x ananassa*** | **Root** | **Leticia** | **N/A** | **N/A** | **PV010607** |
| ***N. rosae*** | **Pe_199_1** | **Feb-2023** | **SPF** | ***Fragaria x ananassa*** | **Crown** | **Leticia** | **N/A** | **N/A** | **PV010608** |
| ***N. rosae*** | **Pe_200_1** | **Feb-2023** | **SPF** | ***Fragaria x ananassa*** | **Crown** | **Leticia** | **N/A** | **N/A** | **PV010609** |
| ***N. rosae*** | **Pe_202_1** | **Feb-2023** | **SPF** | ***Fragaria x ananassa*** | **Crown** | **Leticia** | **N/A** | **N/A** | **PV010610** |
| ***N. rosae*** | **Pe_203_1** | **Feb-2023** | **SPF** | ***Fragaria x ananassa*** | **Crown** | **Leticia** | **N/A** | **N/A** | **PV010611** |
| ***N. rosae*** | **Pe_205_1** | **Feb-2023** | **SPF** | ***Fragaria x ananassa*** | **Crown** | **Leticia** | **N/A** | **N/A** | **PV010612** |
| ***N. rosae*** | **Pe_206_1** | **Feb-2023** | **SPF** | ***Fragaria x ananassa*** | **Crown** | **Leticia** | **N/A** | **N/A** | **PV010613** |
| ***N. rosae*** | **Pe_207_1** | **Feb-2023** | **SPF** | ***Fragaria x ananassa*** | **Crown** | **Leticia** | **N/A** | **N/A** | **PV010614** |
| ***N. rosae*** | **Pe_209_1** | **Feb-2023** | **SPF** | ***Fragaria x ananassa*** | **Crown** | **Leticia** | **N/A** | **N/A** | **PV010615** |
| ***N. rosae*** | **Pe_210_1** | **Feb-2023** | **SPF** | ***Fragaria x ananassa*** | **Leaf** | **Leticia** | **N/A** | **N/A** | **PV010616** |
| ***N. rosae*** | **Pe_211_1** | **Feb-2023** | **SPF** | ***Fragaria x ananassa*** | **Crown** | **Leticia** | **N/A** | **N/A** | **PV010617** |
| ***N. rosae*** | **Pe_216_1** | **Feb-2023** | **SPF** | ***Fragaria x ananassa*** | **Crown** | **Leticia** | **N/A** | **N/A** | **PV010618** |
| ***N. rosae*** | **Pe_217_1** | **Feb-2023** | **SPF** | ***Fragaria x ananassa*** | **Crown** | **Leticia** | **N/A** | **N/A** | **PV010619** |
| ***N. rosae*** | **Pe_218_1** | **Mar-2023** | **NRP (CL)** | ***Fragaria x ananassa*** | **Crown** | **Leticia** | **N/A** | **N/A** | **PV010620** |
| ***N. rosae*** | **Pe_219_1** | **Mar-2023** | **NRP (CL)** | ***Fragaria x ananassa*** | **Crown** | **Leticia** | **N/A** | **N/A** | **PV010621** |
| ***N. rosae*** | **Pe_220_1** | **Mar-2023** | **SPF** | ***Fragaria x ananassa*** | **Crown** | **Leticia** | **N/A** | **N/A** | **PV010622** |
| ***N. rosae*** | **Pe_221_1** | **Mar-2023** | **SPF** | ***Fragaria x ananassa*** | **-** | **Splendor** | **N/A** | **N/A** | **PV010623** |
| ***N. rosae*** | **Pe_223_1** | **Mar-2023** | **SPF** | ***Fragaria x ananassa*** | **Leaf** | **Leticia** | **N/A** | **N/A** | **PV010624** |
| ***N. rosae*** | **Pe_224_1** | **Jun-2023** | **SPF** | ***Fragaria x ananassa*** | **Crown** | **N/A** | **N/A** | **N/A** | **PV010625** |
| ***N. rosae*** | **Pe_225_1** | **Jun-2023** | **SPF** | ***Fragaria x ananassa*** | **Crown** | **N/A** | **N/A** | **N/A** | **PV010626** |
| *N*. sp. | **Pe_14_1** | Nov-2021 | SPF | *Fragaria x ananassa* | N/A | N/A | PQ771736 | PV010452 | PV032029 |
| *N*. sp. | **Pe_32_3** | Jan-2022 | SPF | *Fragaria x ananassa* | N/A | N/A | PQ771737 | PV010453 | PV032030 |
| *N*. sp. | **Pe_35_2** | Jan-2022 | SPF | *Fragaria x ananassa* | N/A | N/A | PQ771738 | PV010454 | PV032031 |
| *N*. sp. | **Pe_36_2** | Jan-2022 | SPF | *Fragaria x ananassa* | N/A | N/A | PQ771739 | PV010455 | PV032032 |
| *N*. sp. | **Pe_36_3** | Jan-2022 | SPF | *Fragaria x ananassa* | N/A | N/A | PQ771740 | PV010456 | PV032033 |
| *N*. sp. | **Pe_37_2** | Jan-2022 | SPF | *Fragaria x ananassa* | N/A | N/A | PQ771741 | PV010457 | PV032034 |
| *N*. sp. | **Pe_37_3** | Jan-2022 | SPF | *Fragaria x ananassa* | N/A | N/A | PQ771742 | PV010458 | PV032035 |
| *N*. sp. | **Pe_50_1** | Feb-2022 | SPF | *Fragaria x ananassa* | Root | Leticia | PQ771743 | PV010459 | PV032036 |
| *N*. sp. | **Pe_56_3** | Apr-2022 | SPF | *Fragaria x ananassa* | Crown | Leticia | PQ771744 | PV010460 | PV032037 |
| *N*. sp. | **Pe_62_1** | Apr-2022 | SPF | *Fragaria x ananassa* | Root | Amparo | PQ771745 | PV010448 | PV032038 |
| *N*. sp. | **Pe_66_1** | Apr-2022 | SPF | *Fragaria x ananassa* | Root | Leticia | PQ771746 | PV010461 | PV032039 |
| *N*. sp. | **Pe_67_1** | Mar-2022 | SPF | *Fragaria x ananassa* | Crown | PALO2B_3 | PQ771747 | PV010462 | PV032040 |
| *N*. sp. | **Pe_69_1** | Mar-2022 | SPF | *Fragaria x ananassa* | Crown | LEO1B_4 | PQ771748 | PV010449 | PV032041 |
| *N*. sp. | **Pe_69_2** | Mar-2022 | SPF | *Fragaria x ananassa* | Crown | LEO1B_4 | PQ771749 | PV010450 | PV032042 |
| *N*. sp. | **Pe_85_1** | Mar-2022 | SPF | *Fragaria x ananassa* | Crown | PALO1B_1 | PQ771750 | PV010463 | PV032043 |
| *N*. sp. | **Pe_85_2** | Mar-2022 | SPF | *Fragaria x ananassa* | Crown | PALO1B_1 | PQ771751 | PV010464 | PV032044 |
| *N*. sp. | **Pe_86_1** | Apr-2022 | SPF | *Fragaria x ananassa* | Crown | Amparo | PQ771752 | PV010465 | PV032045 |
| *N*. sp. | **Pe_102_1** | Jun-2022 | SPF | *Fragaria x ananassa* | Crown | Leticia | PQ771753 | PV010466 | PV032046 |
| *N*. sp. | **Pe_102_2** | Jun-2022 | SPF | *Fragaria x ananassa* | Crown | Leticia | PQ771754 | PV010467 | PV032047 |
| *N*. sp. | **Pe_111_1** | Jun-2022 | SPF | *Fragaria x ananassa* | Root | Palmeritas | PQ771755 | PV010468 | PV032048 |
| *N*. sp. | **Pe_114_2** | Jun-2022 | SPF | *Fragaria x ananassa* | Crown | Palmeritas | PQ771756 | PV010469 | PV032049 |
| *N*. sp. | **Pe_121_2** | Jul-2022 | NRP (G) | *Fragaria x ananassa* | Crown | Leyre | PQ771757 | PV010470 | PV032050 |
| *N*. sp. | **Pe_127_2** | Jul-2022 | NRP (G) | *Fragaria x ananassa* | Crown | Leyre | PQ771758 | PV010471 | PV032051 |
| *N*. sp. | **Pe_129_1** | Jul-2022 | NRP (G) | *Fragaria x ananassa* | Crown | Leyre | PQ771759 | PV010451 | PV032052 |
| *N*. sp. | **Pe_130_1** | Jul-2022 | NRP (G) | *Fragaria x ananassa* | Crown | Leyre | PQ771760 | PV010472 | PV032053 |
| *N*. sp. | **Pe_131_1** | Jul-2022 | NRP (G) | *Fragaria x ananassa* | Crown | Leyre | PQ771761 | PV010473 | PV032054 |
| ***N*****. sp.** | **Pe_135_1** | **Sep-2022** | **NRP (CL)** | ***Fragaria x ananassa*** | **Petiole** | **N/A** | **N/A** | **N/A** | **PV032055** |
| ***N*****. sp.** | **Pe_135_2** | **Sep-2022** | **NRP (CL)** | ***Fragaria x ananassa*** | **Petiole** | **N/A** | **N/A** | **N/A** | **PV032056** |
| ***N*****. sp.** | **Pe_142_1** | **Oct-2022** | **NRP (CL)** | ***Fragaria x ananassa*** | **Leaf** | **N/A** | **N/A** | **N/A** | **PV032057** |
| ***N*****. sp.** | **Pe_143_1** | **Oct-2022** | **NRP (CL)** | ***Fragaria x ananassa*** | **Leaf** | **N/A** | **N/A** | **N/A** | **PV032058** |
| ***N*****. sp.** | **Pe_144_1** | **Oct-2022** | **NRP (CL)** | ***Fragaria x ananassa*** | **Leaf** | **N/A** | **N/A** | **N/A** | **PV032059** |
| ***N*****. sp.** | **Pe_155_1** | **Dec-2022** | **NRP (G)** | ***Fragaria x ananassa*** | **Crown** | **Leticia** | **N/A** | **N/A** | **PV032060** |
| ***N*****. sp.** | **Pe_156_1** | **Dec-2022** | **NRP (CL)** | ***Fragaria x ananassa*** | **Crown** | **Leticia** | **N/A** | **N/A** | **PV032061** |
| ***N*****. sp.** | **Pe_157_1** | **Dec-2022** | **NRP (CL)** | ***Fragaria x ananassa*** | **Root** | **Leticia** | **N/A** | **N/A** | **PV032062** |
| ***N*****. sp.** | **Pe_160_1** | **Dec-2022** | **CD** | ***Fragaria x ananassa*** | **Crown** | **Leticia** | **N/A** | **N/A** | **PV032063** |
| ***N*****. sp.** | **Pe_168_1** | **Dec-2022** | **NRP (G)** | ***Fragaria x ananassa*** | **Root** | **Palmeritas** | **N/A** | **N/A** | **PV032064** |
| ***N*****. sp.** | **Pe_169_1** | **Dec-2022** | **NRP (G)** | ***Fragaria x ananassa*** | **Crown** | **Cleopatra** | **N/A** | **N/A** | **PV032065** |
| ***N*****. sp.** | **Pe_170_1** | **Dec-2022** | **NRP (G)** | ***Fragaria x ananassa*** | **Crown** | **Cleopatra** | **N/A** | **N/A** | **PV032066** |
| ***N*****. sp.** | **Pe_175_1** | **Nov-2022** | **NRP (G)** | ***Fragaria x ananassa*** | **Root** | **Palmeritas** | **N/A** | **N/A** | **PV032067** |
| ***N*****. sp.** | **Pe_178_1** | **Feb-2023** | **SPF** | ***Fragaria x ananassa*** | **Crown** | **Leticia** | **N/A** | **N/A** | **PV032068** |
| ***N*****. sp.** | **Pe_180_1** | **Feb-2023** | **SPF** | ***Fragaria x ananassa*** | **Leaf** | **Leticia** | **N/A** | **N/A** | **PV032069** |
| ***N*****. sp.** | **Pe_191_1** | **Feb-2023** | **SPF** | ***Fragaria x ananassa*** | **Crown** | **Leticia** | **N/A** | **N/A** | **PV032070** |
| ***N*****. sp.** | **Pe_192_1** | **Feb-2023** | **SPF** | ***Fragaria x ananassa*** | **Crown** | **Leticia** | **N/A** | **N/A** | **PV032071** |
| ***N*****. sp.** | **Pe_196_1** | **Feb-2023** | **SPF** | ***Fragaria x ananassa*** | **Crown** | **Leticia** | **N/A** | **N/A** | **PV032072** |
| ***N*****. sp.** | **Pe_201_1** | **Feb-2023** | **SPF** | ***Fragaria x ananassa*** | **Crown** | **Leticia** | **N/A** | **N/A** | **PV032073** |
| ***N*****. sp.** | **Pe_204_1** | **Feb-2023** | **SPF** | ***Fragaria x ananassa*** | **Crown** | **Leticia** | **N/A** | **N/A** | **PV032074** |
| ***N*****. sp.** | **Pe_208_1** | **Feb-2023** | **SPF** | ***Fragaria x ananassa*** | **Crown** | **Leticia** | **N/A** | **N/A** | **PV032075** |
| ***N*****. sp.** | **Pe_212_1** | **Feb-2023** | **SPF** | ***Fragaria x ananassa*** | **Crown** | **Leticia** | **N/A** | **N/A** | **PV032076** |
| ***N*****. sp.** | **Pe_213_1** | **Feb-2023** | **SPF** | ***Fragaria x ananassa*** | **Crown** | **Leticia** | **N/A** | **N/A** | **PV032077** |
| ***N*****. sp.** | **Pe_215_1** | **Feb-2023** | **SPF** | ***Fragaria x ananassa*** | **Root** | **Leticia** | **N/A** | **N/A** | **PV032078** |
| *N. iranensis* | **Pe_17_1** | Nov-2021 | SPF | *Fragaria x ananassa* | N/A | N/A | PQ800741 | PV010474 | PV032079 |
| *N. iranensis* | **Pe_18_1** | Nov-2021 | SPF | *Fragaria x ananassa* | N/A | N/A | PQ800742 | PV032125 | PV032097 |
| *N. iranensis* | **Pe_19_1** | Nov-2021 | SPF | *Fragaria x ananassa* | N/A | N/A | PQ800743 | PV032126 | PV032098 |
| *N. iranensis* | **Pe_21_1** | Nov-2021 | SPF | *Fragaria x ananassa* | N/A | N/A | PQ800744 | PV010475 | PV032080 |
| *N. iranensis* | **Pe_22_1** | Nov-2021 | SPF | *Fragaria x ananassa* | N/A | N/A | PQ800745 | PV010476 | PV032081 |
| *N. iranensis* | **Pe_24_1** | Nov-2021 | NRP (CL) | *Fragaria x ananassa* | N/A | N/A | PQ800746 | PV032127 | PV032100 |
| *N. iranensis* | **Pe_25_1** | Nov-2021 | NRP (CL) | *Fragaria x ananassa* | N/A | N/A | PQ800747 | PV032128 | PV032101 |
| *N. iranensis* | **Pe_27_1** | Nov-2021 | NRP (CL) | *Fragaria x ananassa* | N/A | N/A | PQ800748 | PV032129 | PV032102 |
| *N. iranensis* | **Pe_28_3** | Nov-2021 | NRP (CL) | *Fragaria x ananassa* | N/A | N/A | PQ800749 | PV032130 | PV032103 |
| *N. iranensis* | **Pe_29_2** | Nov-2021 | NRP (CL) | *Fragaria x ananassa* | N/A | N/A | PQ800750 | PV032131 | PV032104 |
| *N. iranensis* | **Pe_30_1** | Nov-2021 | NRP (CL) | *Fragaria x ananassa* | N/A | N/A | PQ800751 | PV032132 | PV032105 |
| *N. iranensis* | **Pe_30_5** | Nov-2021 | NRP (CL) | *Fragaria x ananassa* | N/A | N/A | PQ800752 | PV032133 | PV032106 |
| *N. iranensis* | **Pe_31_1** | Nov-2021 | NRP (CL) | *Fragaria x ananassa* | N/A | N/A | PQ800753 | PV010477 | PV032082 |
| *N. iranensis* | **Pe_39_2** | Jan-2022 | SPF | *Fragaria x ananassa* | N/A | N/A | PQ800754 | PV032134 | PV032107 |
| *N. iranensis* | **Pe_40_1** | Jan-2022 | SPF | *Fragaria x ananassa* | N/A | N/A | PQ800755 | PV032135 | PV032108 |
| *N. iranensis* | **Pe_40_2** | Jan-2022 | SPF | *Fragaria x ananassa* | Crown | Primoris | PQ800756 | PV032136 | PV032109 |
| *N. iranensis* | **Pe_44_1** | Feb-2022 | SPF | *Fragaria x ananassa* | Crown | Primoris | PQ800757 | PV010478 | PV032083 |
| *N. iranensis* | **Pe_46_1** | Feb-2022 | SPF | *Fragaria x ananassa* | Crown | Fortuna | PQ800758 | PV032137 | PV032084 |
| *N. iranensis* | **Pe_47_1** | Feb-2022 | SPF | *Fragaria x ananassa* | Crown | Fortuna | PQ800759 | PV032138 | PV032110 |
| *N. iranensis* | **Pe_48_1** | Feb-2022 | SPF | *Fragaria x ananassa* | Crown | Fortuna | PQ800760 | PV032139 | PV032111 |
| *N. iranensis* | **Pe_49_1** | Feb-2022 | SPF | *Fragaria x ananassa* | Crown | Leticia | PQ800761 | PV010479 | PV032085 |
| *N. iranensis* | **Pe_52_1** | Feb-2022 | SPF | *Fragaria x ananassa* | Crown | Leticia | PQ800762 | PV010480 | PV032086 |
| *N. iranensis* | **Pe_52_2** | Feb-2022 | SPF | *Fragaria x ananassa* | Crown | Leticia | PQ800763 | PV010481 | PV032087 |
| *N. iranensis* | **Pe_72_1** | Apr-2022 | SPF | *Fragaria x ananassa* | Petiole | VK-19-138 | PQ800764 | PV032140 | PV032112 |
| *N. iranensis* | **Pe_79_2** | Apr-2022 | SPF | *Fragaria x ananassa* | Crown | Calinda | PQ800765 | PV032141 | PV032113 |
| *N. iranensis* | **Pe_81_1** | Apr-2022 | SPF | *Fragaria x ananassa* | Crown | Calinda | PQ800766 | PV032142 | PV032114 |
| *N. iranensis* | **Pe_82_1** | Apr-2022 | SPF | *Fragaria x ananassa* | Crown | Calinda | PQ800767 | PV032143 | PV032115 |
| *N. iranensis* | **Pe_82_2** | Apr-2022 | SPF | *Fragaria x ananassa* | Crown | Calinda | PQ800768 | PV032144 | PV032116 |
| *N. iranensis* | **Pe_83_1** | Apr-2022 | SPF | *Fragaria x ananassa* | Crown | Calinda | PQ800769 | PV010482 | PV032088 |
| *N. iranensis* | **Pe_84_2** | Apr-2022 | SPF | *Fragaria x ananassa* | Crown | Calinda | PQ800770 | PV032145 | PV032117 |
| *N. iranensis* | **Pe_87_1** | Apr-2022 | SPF | *Fragaria x ananassa* | Crown | vK-19-138 | PQ800771 | PV010483 | PV032089 |
| *N. iranensis* | **Pe_92_1** | May-2022 | SPF | *Fragaria x ananassa* | Crown | Leticia | PQ800772 | PV032146 | PV032118 |
| *N. iranensis* | **Pe_93_1** | May-2022 | SPF | *Fragaria x ananassa* | Crown | RISEC | PQ800773 | PV032147 | PV032119 |
| *N. iranensis* | **Pe_94_3** | May-2022 | SPF | *Fragaria x ananassa* | Crown | RISEC | PQ800774 | PV032148 | PV032120 |
| *N. iranensis* | **Pe_95_1** | Apr-2022 | SPF | *Fragaria x ananassa* | Root | Leyre | PQ800775 | PV010484 | PV032090 |
| *N. iranensis* | **Pe_96_1** | May-2022 | SPF | *Fragaria x ananassa* | Crown | Candy FNM | PQ800776 | PV010485 | PV032091 |
| *N. iranensis* | **Pe_97_1** | May-2022 | SPF | *Fragaria x ananassa* | Crown | Candy FNM | PQ800777 | PV010486 | PV032092 |
| *N. iranensis* | **Pe_98_2** | May-2022 | SPF | *Fragaria x ananassa* | Crown | Candy FNM | PQ800778 | PV010487 | PV032093 |
| *N. iranensis* | **Pe_99_1** | May-2022 | SPF | *Fragaria x ananassa* | Crown | RISEC | PQ800779 | PV032149 | PV032121 |
| *N. iranensis* | **Pe_100_1** | May-2022 | SPF | *Fragaria x ananassa* | Root | RIVRE | PQ800780 | PV010488 | PV032094 |
| ***N. iranensis*** | **Pe_146_1** | **Oct-2022** | **NRP (CL)** | ***Fragaria x ananassa*** | **Crown** | **Beauty** | **N/A** | **N/A** | **PV032095** |
| ***N. iranensis*** | **Pe_148_1** | **Oct-2022** | **NRP (CL)** | ***Fragaria x ananassa*** | **Crown** | **Beauty** | **N/A** | **N/A** | **PV032122** |
| ***N. iranensis*** | **Pe_153_1** | **Sep-2022** | **CD** | ***Fragaria x ananassa*** | **Crown** | **Leyre** | **N/A** | **N/A** | **PV032096** |
| ***N. iranensis*** | **Pe_164_1** | **Dec-2022** | **NRP (CL)** | ***Fragaria x ananassa*** | **Crown** | **Fortuna** | **N/A** | **N/A** | **PV032123** |
| ***N. iranensis*** | **Pe_222_1** | **Mar-2023** | **SPF** | ***Fragaria x ananassa*** | **Leaf** | **Leticia** | **N/A** | **N/A** | **PV032124** |

^q ^*Neopestalotiopsis* species. 

^r ^Isolate codes of the mycological library in the Phytopathology laboratory at the E.T.S.I.A. of University of Seville.

^s ^Date when symptomatic plants were collected. In 2021-2022 season, twenty-six plots were sampled in the municipalities of Palos de la Frontera, Moguer, Lepe, Isla Cristina, Villablanca, Gibraleón and Almonte. In 2022-2023 season, six plots were sampled in the municipalities of Palos de la Frontera, Lepe, Villablanca, and Almonte. 

^t ^Place where the isolate was recovered. SPF: Strawberry production fields from Huelva province in southwestern Spain. BPF: Blueberry production fields from Huelva province in southwestern Spain NRP: NRP runner plant. Nurseries were located at Segovia and Ávila provinces in Castilla y Leon (CL) in northern Spain and Granada (G) province in southern Spain. CD: Crown debris that spent the summer in strawberry production fields at Huelva.

^u ^Host from where the isolate was recovered.

^v ^Tissue from which the isolate was recovered.

^w ^Cultivar from which the isolate was recovered.

^x^ GenBank accession number for the internal transcribed spacer (ITS) region.

^y^ GenBank accession number for the translation elongation factor (TEF) region.

^z ^GenBank accession number for the beta-tubulin (TUB) region.

N/A: reference not available.

* Rows in bold mean that these isolates are from the 2022-2023 strawberry season. They appear in the phylogenetic tree conducted by using only TUB sequences (Figure S1).
