# Supplementary material for: Molecular characterization reveals three Neopestalotiopsis species causing strawberry disease outbreaks in Spain
Source: Front Plant Sci. 2026 May 11;17:1830265. doi: 10.3389/fpls.2026.1830265 (PMC13199264; doi:10.3389/fpls.2026.1830265)
Supplement: Supplementary file 7 [file Table2.docx]

Table S2. Analysis of variance results for disease incidence for foliar and crown inoculation.

| Source | foliar inoculation |  |  |  | crown inoculation |  |  |
| --- | --- | --- | --- | --- | --- | --- | --- |
|  | DF^a^ | MS^b^ | P-value |  | DF | MS | P-value |
| Isolate | 5 | 0.190 | 0.0091 |  | 5 | 0.244 | 0.3751 |
| Cultivar | 2 | 0.654 | 0.0002 |  | 2 | 0.341 | 0.0385 |
| Assay | 1 | 0.010 | 0.6356 |  | 1 | 0.142 | 0.0854 |
| Isolate x Cultivar | 10 | 0.053 | 0.3364 |  | 10 | 0.376 | 0.5639 |
| Error | 17 | 0.043 |  |  | 17 | 1.851 |  |

^a^: DF: degrees of freedom. ^b^: MS: mean square.

The data did not need to be transformed to meet the requirements of ANOVA.
