# Supplementary material for: Molecular characterization reveals three Neopestalotiopsis species causing strawberry disease outbreaks in Spain
Source: Front Plant Sci. 2026 May 11;17:1830265. doi: 10.3389/fpls.2026.1830265 (PMC13199264; doi:10.3389/fpls.2026.1830265)
Supplement: Supplementary file 8 [file Table3.docx]

Table S3. Analysis of variance results for disease severity for foliar and crown inoculation.

| Source | foliar inoculation |  |  |  | crown inoculation |  |  |
| --- | --- | --- | --- | --- | --- | --- | --- |
|  | DF^a^ | MS^b^ | P-value |  | DF | MS | P-value |
| Isolate | 5 | 0.156 | 0.0000 |  | 5 | 4.479 | 0.7243 |
| Cultivar | 2 | 0.131 | 0.0039 |  | 2 | 3.617 | 0.6330 |
| Assay | 1 | 0.005 | 0.7283 |  | 1 | 27.62 | 0.0716 |
| Blok (Assay) | 8 | 0.042 | 0.0724 |  | 8 | 6.415 | 0.5917 |
| Isolate x Cultivar | 10 | 0.028 | 0.273 |  | 10 | 8.584 | 0.3743 |
| Error | 153 | 0.023 |  |  | 153 | 7.885 |  |

^a^: DF: degrees of freedom. ^b^: MS: mean square.

To satisfy the assumptions of normality and homogeneity of variance, the data on severity following leaf inoculation were transformed using the square root, whilst the data on severity following crown inoculation were transformed using the logarithm prior to statistical analysis.
